# Phenotyping of *Drosophila Melanogaster*—A Nutritional Perspective

**DOI:** 10.3390/biom12020221

**Published:** 2022-01-27

**Authors:** Virginia Eickelberg, Kai Lüersen, Stefanie Staats, Gerald Rimbach

**Affiliations:** Department of Food Science, Institute of Human Nutrition and Food Science, University of Kiel, Hermann-Rodewald-Strasse 6-8, D-24118 Kiel, Germany; luersen@foodsci.uni-kiel.de (K.L.); staats@foodsci.uni-kiel.de (S.S.); rimbach@foodsci.uni-kiel.de (G.R.)

**Keywords:** *Drosophila melanogaster*, high-sugar diet, high-fat diet, obesity, phenotyping

## Abstract

The model organism *Drosophila melanogaster* was increasingly applied in nutrition research in recent years. A range of methods are available for the phenotyping of *D. melanogaster*, which are outlined in the first part of this review. The methods include determinations of body weight, body composition, food intake, lifespan, locomotor activity, reproductive capacity and stress tolerance. In the second part, the practical application of the phenotyping of flies is demonstrated via a discussion of obese phenotypes in response to high-sugar diet (HSD) and high-fat diet (HFD) feeding. HSD feeding and HFD feeding are dietary interventions that lead to an increase in fat storage and affect carbohydrate-insulin homeostasis, lifespan, locomotor activity, reproductive capacity and stress tolerance. Furthermore, studies regarding the impacts of HSD and HFD on the transcriptome and metabolome of *D. melanogaster* are important for relating phenotypic changes to underlying molecular mechanisms. Overall, *D. melanogaster* was demonstrated to be a valuable model organism with which to examine the pathogeneses and underlying molecular mechanisms of common chronic metabolic diseases in a nutritional context.

## 1. Introduction

Since the early 20th century, the fruit fly, *Drosophila melanogaster*, has been used as an important model organism in experimental research. The use of *D. melanogaster* provides several advantages. First, the number of offspring per fertilized female is relatively large, while the life cycle is short. The developmental time from egg to hatched fly is, on average, nine to ten days at 25 °C [1]. This ensures the availability of a sufficient number of fruit flies for research in a short period of time. Second, the cultivation and maintenance of *D. melanogaster* in large numbers are practicable, and the costs are moderate. Furthermore, *D. melanogaster* has a comparatively short lifespan, with a maximum lifespan of approximately 60 days for males and 80 days for females depending on the holding conditions and strains, which allows lifelong examinations. Further advantages are that the genome of *D. melanogaster* is completely sequenced [2] and that nearly 75% of the disease-related genes in humans have functional orthologous genes in *D. melanogaster* [3].

Common fields of application for *D. melanogaster* as a model organism are genetics, developmental biology and biomedicine. Specific mutants and transgenic flies can be used as models that mimic various chronic diseases [4,5,6,7].

In recent years, the value of *D. melanogaster* in nutrition research became increasingly recognized. In this context, the roles of different dietary compounds, diet compositions and plant bioactive components on various readouts were investigated [8,9,10,11,12]. As a standard diet, a complex medium comprising yeast, sugar and agar is commonly used, which can be easily and rapidly prepared, providing all of the necessary nutrients to flies [4]. However, to unravel the influence of distinct dietary factors, semidefined or fully defined diets, such as holidic media, represent helpful alternatives [13].

A variety of methods can be applied for the phenotyping of *D. melanogaster*. Important readouts in nutritional studies are measurements of food intake, body weight and composition, as well as metabolic and digestive function. Other important phenotypes are lifespan, fitness, locomotor activity, responses to environmental stresses and reproductive and developmental capacity [4,14].

In the first part of this review, methods for phenotyping *D. melanogaster* in a nutritional context are outlined, and the advantages and disadvantages of the different methods are illustrated. In the second part, the application of comprehensive phenotyping is demonstrated with examples of dietary interventions, namely high-sugar diet (HSD) and high-fat diet (HFD) feeding. Finally, studies regarding the impacts of HSD and HFD feeding on the transcriptome and metabolome of *D. melanogaster* are presented, which place the nutritional phenotypes in the context of underlying cellular and molecular mechanisms.

## 2. Phenotyping of *D. melanogaster*

### 2.1. Body Weight

An important step in nutrition research in flies was made by Mackay and coworkers through the quantification of genetic variation in body weight and major metabolites (such as glycogen, triacylglyceride (TAG)) and their association with obesity and the metabolic rate in flies [15]. For the determination of body weight, flies have to be sex-specifically pooled after prefeeding. Following transfer to sealable vials, groups of at least 10–20 animals are weighed with a precision scale, and the average body weight per fly is calculated [14]. Additionally, the dry mass of *D. melanogaster* can be quantified. To this end, the fruit flies are dried in an oven (e.g., at 60 °C for 24 h) before their body mass is determined with a precision scale [16]. By calculating the difference between wet and dry weight, the water content of the flies can be assessed.

### 2.2. Body Composition: Lipids, Proteins, Carbohydrates

#### 2.2.1. Lipid Determination

An overview of the different methods for the determination of fat content, with advantages and disadvantages, is provided in Table 1. Body fat in *D. melanogaster* consists mainly of TAGs, which are stored in lipid droplets of adipocytes [17]. The number and size of lipid droplets differ depending on the type of tissue and environmental conditions, such as the ingested diet. Lipid droplets can be evaluated and quantified visually by staining with dyes such as Nile red, BODIPY or Harris hematoxylin [18,19,20]. This method is applicable for examination of tissues at the cellular level but is limited in the quantification of the total TAG content of adult flies due to the different fat-accumulating organs, such as the fat body, intestine and ovaries [17].

**Figure 1:**
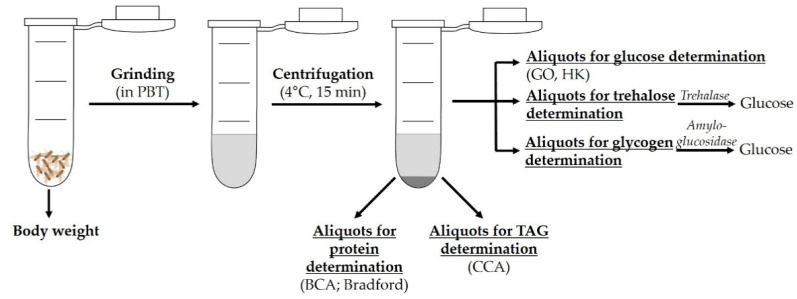
Exemplary protocol for assessment of the body composition of *Drosophila melanogaster*. After determining their body weight, flies are ground in Dulbecco’s phosphate-buffered saline with Triton X-100 (PBT) by using a tissue homogenizer. Following centrifugation, aliquots of the supernatant are utilized for further measurements. The protein content is determined with a bicinchoninic acid (BCA) or Bradford assay, the TAG content with coupled colorimetric assays (CCAs) and the glucose content with enzymatic methods based on the enzyme glucose oxidase (GO) or hexokinase (HK). For assessment of trehalose and glycogen concentrations, enzymatic treatments with trehalase and amyloglucosidase are conducted, respectively, after which free glucose is determined (modified from [28]).

To determine whole-body TAG content, a fly homogenate is initially made via grinding of the flies in a buffer solution and subsequent centrifugation (Figure 1). Commonly applied methods for TAG measurement are coupled colorimetric assays (CCAs), for which kits from various manufacturers are available. CCAs are generally based on the same reaction scheme, which starts with the enzymatic hydrolysis of TAGs by lipoprotein lipase into glycerol and free fatty acids (FA) [17,21,22,23,24]. The glycerol is then enzymatically phosphorylated and oxidized, generating hydrogen peroxide that forms a red quinoneimine dye with 4-aminoantipyrine and 3,5-dichloro-2-hydroxybenzene sulfonate in the last reaction step. The absorption of this red dye is proportional to the TAG concentration of the sample [25,26]. These CCAs enable the rapid determination of the TAG content with a large sample size. However, owing to the insolubility of the stored fats in adipocytes of *D. melanogaster*, the accuracy of the determination of absolute TAG levels with CCAs is questioned. Additionally, the identification of the proportions of different glycerides in the initial sample is not possible because of the determination of the total glycerol content at the final reaction step. However, the free glycerol present in flies is reported to be neglectable [17]. It should also be noted that the colored eye pigments of *D. melanogaster* can distort the results, so removal of the heads prior to measurement is recommended [27].

**Figure 2:**
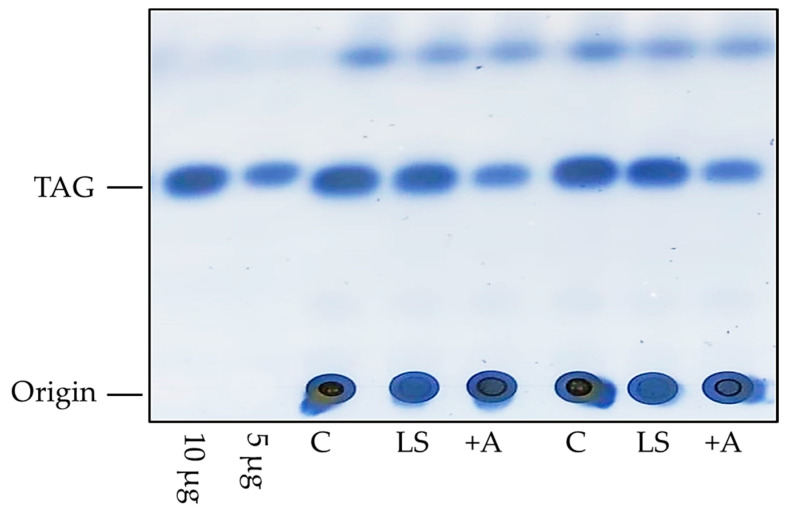
Example of triacylglyceride (TAG) determination in *Drosophila melanogaster* by thin-layer chromatography (TLC). Starting with eggs, fruit flies were raised on different experimental diets, and females were harvested 10 days after eclosion. TAGs were extracted from the flies, separated by TLC and stained according to the methods of Al-Anzi et al. (2009) [31]. A low-starch diet (LS) and acarbose treatment (+A) led to decreased TAG levels. C, control diet containing 10% starch; LS, low-sugar diet containing 1% starch; +A, control diet supplemented with 1.8 mg/L acarbose. Lard was used as a standard (the two bands on the left).

Another method for lipid quantification in *D. melanogaster* is thin-layer chromatography (TLC, Figure 2). The extraction of lipids with a mixture of two or three organic solvents, such as chloroform or methanol, in different mass ratios is necessary for TLC. The extraction techniques of Bligh and Dyer [29] or Folch [30] are appropriate for *D. melanogaster*. The extracted lipid samples and lipid standards are separated on a plastic or glass TLC plate coated with silica (stationary phase), and the liquid (mobile) phase is hexane, ethyl ether and acetic acid. The TLC plate is then air-dried and stained with, e.g., copper (II) sulfate pentahydrate or ceric ammonium molybdate solution depending on the fat group for detection. With TLC, the proportions of TAGs, diglycerides and FAs in the sample can be measured. In addition, quantification of the lipid classes by the application of photodensitometry is possible [17,31,32].

For the determination and quantification of different lipid types, a more specific and highly sensitive analytical method is needed. For this purpose, gas chromatography/mass spectrometry (GC/MS) or liquid chromatography/mass spectrometry (LC/MS) are suitable. After extraction (e.g., according to the methods of Bligh and Dyer [29] or Folch [30]), samples are dissolved in a mobile phase such as ammonium acetate in methanol/chloroform or methanol/water and injected onto a silica column [33,34,35]. Although a precise analysis of lipids is possible with GC/MS or LC/MS, a disadvantage lies in the relatively high cost per measurement.

#### 2.2.2. Protein Determination

Whole-body protein content of *D. melanogaster* is usually determined by assays based on colorimetric or fluorescent signals. The supplies can be obtained as test kits from various commercial suppliers. In these assays, the protein content is measured in the supernatant of a sample after homogenization and centrifugation (Figure 1). The two most common methods for protein determination are the Bradford assay and the bicinchoninic acid (BCA) assay. Both are colorimetric assays, feasible in test-tube or microplate format, using bovine serum albumin (BSA) as a standard protein [36,37,38].

The Bradford assay can be applied rapidly and simply at room temperature with only a minimal and tolerable influence of substances such as thiols, metal chelators or reducing agents. However, one must keep in mind that the measurement might be affected by surfactants such as Triton X-100 or a strong alkaline buffer, which can cause precipitation. Thus, the concentrations of these substances must be considered and adjusted if necessary [36,37].

The BCA assay is also rapid and simple to perform. Fewer protein–protein variations occur in the BCA assay than in the Bradford assay. Additionally, many surfactants can be applied without adverse effects on the BCA assay outcome. However, a multiplicity of substances in solvents or buffers (e.g., ascorbic acid, iron) have copper-reducing or chelating capabilities, which could influence the test results. The removal of these interfering substances is mandatory for precise protein determination [37,38].

#### 2.2.3. Carbohydrate Determination

For the phenotyping of *D. melanogaster*, the determination of glucose, trehalose and glycogen could also be of importance. Depending on the research objectives and parameters, such as the developmental stage of the fly, free glucose can be measured in whole-body homogenates or in hemolymph. For the determination of glucose in whole-body homogenates, flies are initially ground in PBT and centrifuged, and the supernatant is subsequently used for a glucose assay (Figure 1). The measurement of the whole-body homogenate can be rapidly conducted, and the sample throughput is high. However, the intestinal glucose content, derived from the diet, could affect the determination. Additionally, the differentiation between circulating and stored glucose concentrations cannot be made due to the continuous exchange between intracellular and extracellular components; thus, only an approximate content of free glucose can be obtained [39].

A precise measurement of free circulating glucose is possible via the quantification of glucose in the hemolymph of the fly, which is collected from the head or thorax by capillary action or puncture with subsequent centrifugation [40].

Two common enzymatic methods are available to measure free glucose in the hemolymph or in whole-body homogenate. In the first method, the enzyme glucose oxidase (GO) is used to catalyze the oxidation of glucose via the production of gluconic acid and hydrogen peroxide. Subsequently, hydrogen peroxide reacts with various chromophores in combination with peroxidase to produce a colored product that is photometrically or fluorometrically assessed, enabling the calculation of the glucose concentration [41,42].

In the second method, D-glucose is converted by two consecutive enzymatic steps into gluconate-6-phosphate, resulting in the formation of reduced nicotinamide-adenine dinucleotide phosphate (NADPH), which is measured spectrophotometrically. The amount of NADPH synthesized is stoichiometrically related to the glucose concentration of the sample [39,42].

For measurement of trehalose, the disaccharide is initially split by the enzyme trehalase into two D-glucose monomers, which can be assessed enzymatically as described above.

Glycogen in adult flies is primarily located in the fat body, muscle tissue and intestine and can be visualized by periodic acid–Schiff staining or quantified by enzymatic methods. For this, the sample is incubated with the enzyme amyloglucosidase, which cleaves glycogen into free glucose, which then can be measured as described above [28].

### 2.3. Metabolic Rate

The metabolic rate reveals information about health and fitness status and is influenced by factors such as environmental temperature [43], group interactions [44], age, physical activity and diet [45]. In recent publications, CO_2_ measurements via respirometry were introduced in *Drosophila* research, leading primarily to the identification of sensory perception, which induced aversive cues and modulates lifespan through serotonergic signaling [46]. The metabolic rate of *D. melanogaster* is assessed by either direct or indirect calorimetric measurements.

For direct calorimetric measurement, the flies are placed in a calorimetric experimental chamber with a sensitive thermostat, where the heat produced by the organism is determined. The metabolic rates of different flies can be recorded in parallel in real time. To increase the informative value, the measurement can be completed by recording the fly’s movements [45]. The direct calorimetric determination of metabolic rate enables the assessment of aerobic and anaerobic catabolism [47]. A disadvantage of this direct technique is the requirement of specific, mostly custom-built equipment.

During indirect calorimetric determination (respirometry), flies are placed in a sealed experimental chamber with flow-through and optional flushing with CO_2_-free room air. The changes in the gas concentration in the chamber are measured with a gas analyzer, and the oxygen (O_2_) consumption and/or CO_2_ production per time interval is inferred. The metabolic rate of *D. melanogaster* is calculated based on the respiratory quotient as the ratio of produced CO_2_ to consumed O_2_ [48,49].

**Figure 3:**
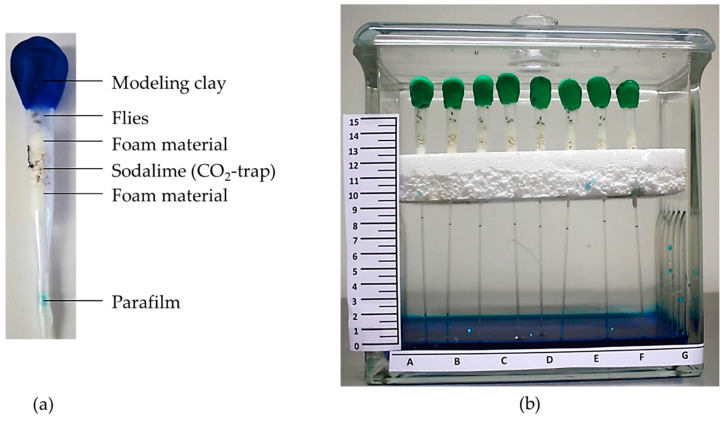
Schematic of the self-assembled respirometer setup. (**a**) The respirometer is a small metabolic chamber built from a pipette tip, a micropipette, Parafilm, foam and CO_2_-absorbing soda lime. After brief anesthetization, flies are placed into the respirometer chamber, which is subsequently closed with a plug of food and covered with plasticine. (**b**) After an adaptation period, the respirometers are inserted into a holder in an outer chamber so that the micropipette tips are immersed into a layer of dyed water. The lid of the outer chamber is closed, and a first photograph is taken. The changes in the filling volume of the micropipettes are documented with additional photographs. By using image processing software, the distances of capillary filling are determined. The amount of CO_2_ produced per fly and hour is calculated via the formula: (micropipette radius × ratio of capillary filling of the micropipette at the start and end point for the sample—negative control) × 1000)/(number of flies × duration of exposure) (modified from [43]).

As an alternative to complex measuring instruments, the amount of produced CO_2_ can be determined using a self-assembled respirometer, which is built from a pipette tip, micropipette and CO_2_-absorbing soda lime (Figure 3). The flies, which are inserted in the respirometer, convert O_2_ into CO_2_. Since CO_2_ is trapped by soda lime, the gas volume in the chamber will decrease over time. This can be monitored by the rising level of the dyed water in the connected micropipettes. By using image processing software, the distances of the capillary filling within a certain period of time are determined to calculate the amount of CO_2_ produced per fly and time. This method is simple and cost-effective, and the metabolic rate of flies from different treatments can be studied simultaneously [43]. However, the assumption in respirometry of approximate values for the correlation between gas exchange and metabolic substrate utilization, especially in smaller organisms, leads to a high error rate for the indirect caloric determination of metabolic rate compared to direct measurements. Additionally, the metabolic rate can be estimated by indirect calorimetry only for aerobic catabolism [47].

### 2.4. Food Intake

**Figure 4:**
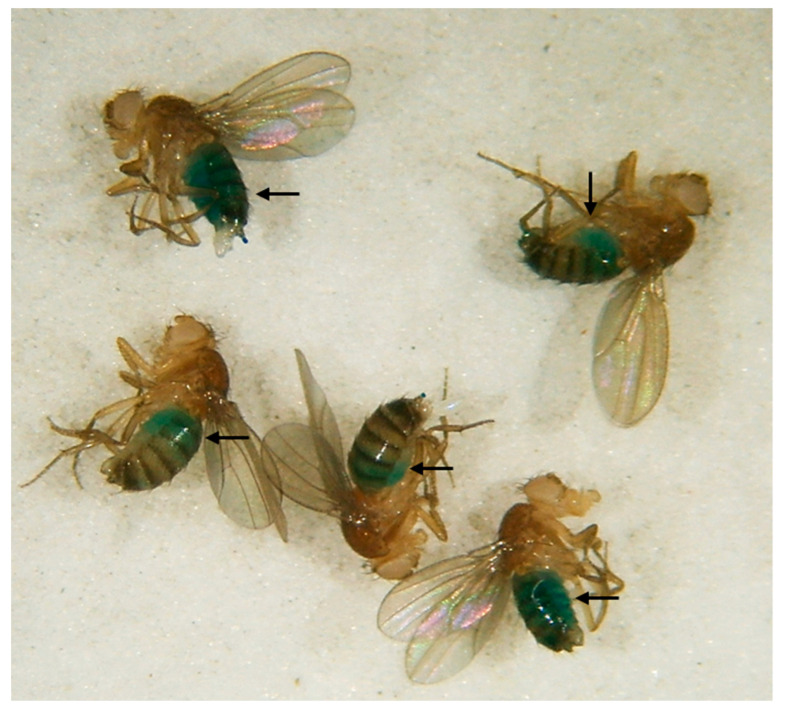
Determination of food intake with dyed medium. Flies are fed on medium supplemented with the nonabsorbable dye, Brilliant Blue FCF. After a defined period of time, the animals are removed, and dye accumulation within the digestive tract is indicated via assessment of the blue-colored abdomen (black arrows). The amount of ingested dye can be determined in the fly lysate by a spectrophotometer. The background absorption of fly lysates must be considered (modified from [10]).

In the following section, several methods for the indirect determination of food intake are introduced. One option for food intake monitoring is the use of trackable supplements such as radioisotope-labeled metabolites or nonabsorbable dyes (Brilliant Blue FCF or Sulforhodamine B) (Figure 4) [10,50,51,52,53]. In the case of dyes, flies are fed on the labeled medium for a defined period of time. Subsequently, the amount of dye that has accumulated in the digestive tract, which reflects food intake, is quantified in fly lysates by using a spectrophotometer or fluorescence reader. In this method, it is important to consider the background pigmentation of the fly lysates. Although simple, this dye accumulation method is thought to have several pitfalls. Most importantly, the incubation time is critical since the linearity of accumulation ends when excretion of the supplement begins. Hence, intake rates can be determined solely during the short (<30 min) initial phase of feeding, and long-term measurement of food intake becomes inaccurate [54]. However, short feeding periods generate substantial biological variability. Furthermore, flies have to be sacrificed, making it impossible to monitor the food intake of a particular fly population over time.

To circumvent the limitations of dye accumulation assays, a consumption-excretion (Con-Ex) method was recently established, which enables the long-term measurement of food intake. Flies are transferred to an empty vial containing a colored medium in a small feeder cup in the lid. After a defined feeding period, the ingested dye content is determined by homogenization of the flies. Additionally, the excreted dye, which is spread on the walls of the vials, is collected, and the absorption of the collected dye fractions (fly lysate and dissolved excreta) is measured by a spectrophotometer. Food intake is calculated using a standard curve [55].

**Figure 5:**
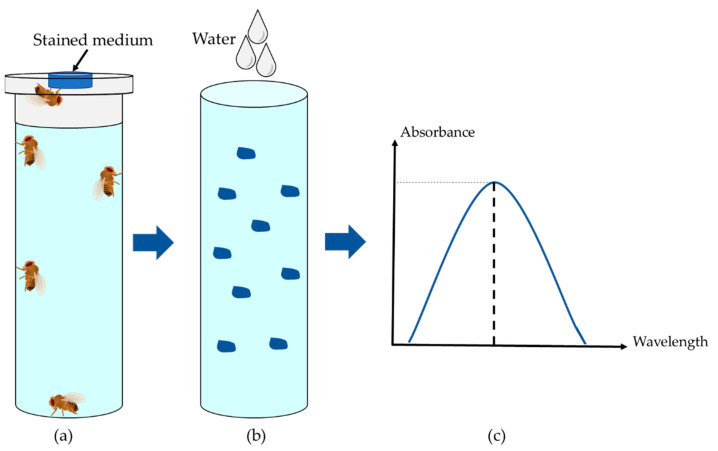
Excreta quantification (EX-Q). (**a**) For EX-Q, flies ingest a medium stained with a nonabsorbent dye in the overhead position, and the dye is excreted with the feces. (**b**) The flies are removed, and the vials with the fecal spots are rinsed with water. (**c**) The absorption is measured photometrically, and the amount of ingested food is quantified with a standard curve (modified from [56]).

Excreta quantification (EX-Q), a modification of the Con-Ex method also enables the determination of food intake without fly sacrifice (Figure 5). A reduction in the surface of the feeder cap enables the quantification of the complete food intake using the excretions [56].

As an alternative to dyes, the medium can be supplemented with radioactively labeled substances such as [^14^C]-choline, [^3^H]-thymidine or [α-32P]-dCTP [57,58,59,60,61,62,63]. Similar to the methods using dyed medium, flies are fed on labeled medium for a certain period of time. In most cases, the ingested food volume is quantified with liquid scintillation counting. However, radioisotopes such as [α-32P] can be measured through the cuticle so that the homogenization of flies is not necessary. This method is described as very reliable and sensitive. However, radioactive-labeled metabolites differ in their adsorption rates in the digestive tract, which can lead to retention of radioisotopes, leading to the underestimation of food intake [64].

**Figure 6:**
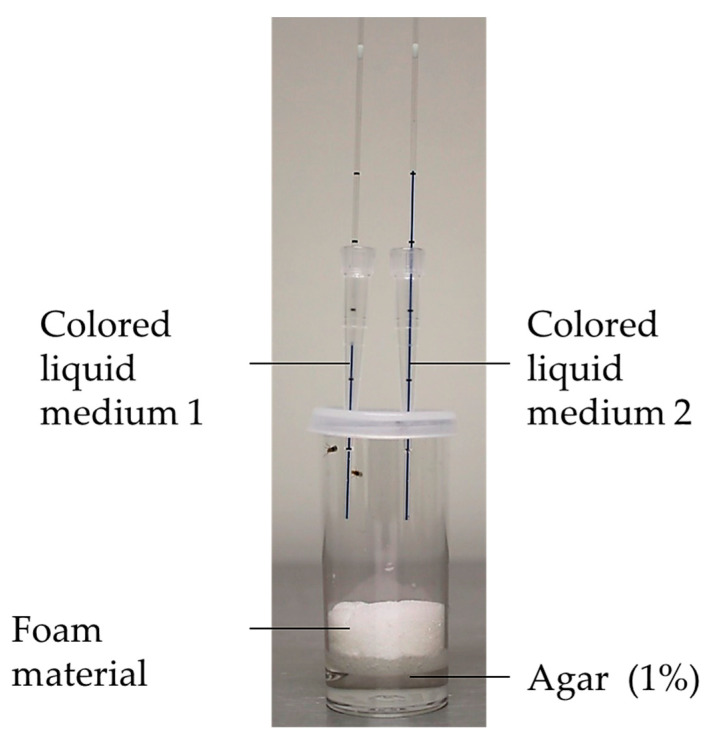
CApillary FEeder (CAFE) assay. The CAFE assay can be used for quantification of food intake and for food choice experiments. Flies are transferred into a vial, which is closed with a lid with small air holes. Capillaries filled with liquid medium and placed into pipette tips are inserted vertically through these air holes. Subsequently, the vials are kept in a storage box to maintain a constant humidity level. The flies absorb the liquid food by feeding on the lower ends of the capillaries. As time passes, the fill volumes of the capillaries decrease. The amount of ingested food can be calculated by measuring the fill levels of each capillary at the start and end of the CAFE assay (modified from [65,66]).

The direct measurement of food intake can be obtained by the CApillary FEeder (CAFE) assay (Figure 6) [65,66]. In this assay, flies are kept in vials under constant humidity. Capillaries containing the liquid medium are placed vertically through the lids of the vials. Flies absorb the liquid by feeding on the ends of the capillaries. Consequently, by measuring the drop-in volume in the capillary, the amount of food intake can be determined. This assay can be used for quantification of ingested food, as well as for choice experiments. An advantage of the CAFE assay is the direct measurement of food intake without the need to anesthetize or kill flies, which enables the long-term monitoring of food intake under different conditions (e.g., lifespan experiments). The CAFE assay is described as an accurate and consistent method [64], whereas it is not suitable for all fly strains in view of the overhead position of the food source, since flies with mobility impairments are unable to reach the feeding place. In addition, only liquid media, and not the commonly used agar-based medium, can be applied in this assay [56]. Garlapow and coworkers performed genome-wide association studies for food intake and environmental variance of food intake, which resulted in the identification of numerous novel genes, related to feeding behavior [67].

For the MAnual FEeding (MAFE) assay, individual flies are carefully aspirated into a pipette tip following a starvation phase. A capillary with a liquid medium is placed on the proboscis of the fly, and the complete extension of the proboscis is monitored optically until there is no additional reaction to the feeding stimulus. The ingested volume and the duration of the food intake are recorded. The food intake of the individual fly can be recorded on a scale of seconds [68]. The MAFE assay should be performed by the same person each time, as the results strongly depend on the handling by the experimenter (e.g., position of the fly in the pipette tip). Additionally, this assay only works in starved flies and requires a high expenditure of time per application, which means that this assay only suitable for a small number of test species [68,69].

The fly Proboscis and Activity Detector (flyPAD) is an automated tool used to sensitively analyze eating behavior and food choice experiments with very small amounts of food. In this method, flies are placed, after a short starvation period, in a behavioral arena with two independent channels containing a solid medium. By contacting the food surface with the proboscis, a change in capacity is induced, which results in an electronic signal. The simultaneous recording of multiple behavioral arenas is possible; however, it is not suitable for large-scale experiments. Furthermore, the long-term recording of food intake is not practicable with the flyPAD [70].

The Fly Liquid-Food Interaction Counter (FLIC) is an automatic tool used to record food volume and choice via the continuous recording of the number and duration of each interaction of the fly (or group of flies) with a liquid medium. This allows for the recording of data on movement and the circadian feeding behavior of flies for short- and long-term periods [71]. The system can be extended with further phenotypic elements such as shock triggering, which enables the examination of food-related learning processes.

The advantage of automated techniques lies in the standardization and independence of the experimenter; however, they are relatively cost-intensive.

The introduction of different methods for the determination of food intake shows the diversity of the methods. Each method offers merits and drawbacks and should be chosen according to the research purpose (Table 2). To increase the reliability of the results, it is useful to validate the results with an additional method.

### 2.5. Lifespan

**Figure 7:**
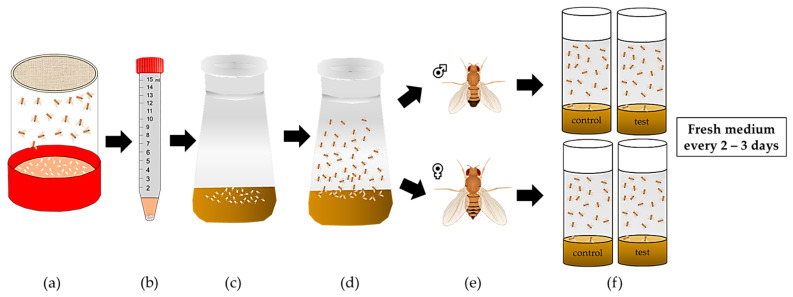
Implementation of a lifespan experiment. (**a**) Adult flies are transferred into an egg-laying cage covered by a grape agar plate with active yeast paste. After 24 h, the grape agar plate is replaced and inoculated with only a small spot of active yeast paste. (**b**) The next day, flies are removed from the cage, and the laid eggs are removed from the surface of the plate with a cotton swab and a physiological buffer or saline solution. The egg suspension is rinsed three times with buffer or saline solution before (**c**) aliquots of the egg suspension are transferred to cultivation medium, where egg-to-adult fly development occurs within approximately 10 days. (**d**) After hatching, the mixed fly populations are transferred to fresh standard medium for mating. (**e**) Forty-eight hours later, the adult flies are usually separated into male and female cohorts. (**f**) Approximately 20 to 30 flies per sex are placed in a vial with a control or experimental medium. Four to five replicates are used to obtain a total number of at least 100 flies per sex and treatment. The flies are transferred to fresh medium every 2 to 3 days, and the number of dead flies is documented at each transfer. This documentation is continued until all flies are dead (modified from [72,73]).

For lifespan experiments, *D. melanogaster* cultures need to be initially synchronized (Figure 7). To this end, freshly laid eggs are collected from the plate using a buffer or a saline solution, rinsed several times and transferred to cultivation medium until hatching. A total of 100 age-matched flies per sex and condition are allocated to an appropriate number of experimental vials, and their survival is monitored and documented over time until the death of the last fly [72,73]. The flies are transferred to a vial with fresh medium every two to three days.

To investigate the long-term impact of different nutritional factors on the survival of fruit flies, lifespan experiments can be conducted. An approach to combining genetic traits with longevity and ageing was undertaken by the Pletcher lab [74,75] through the examination of environmentally induced heterogeneity and its influence on age-specific genetic variance for mortality rates. Furthermore, the group applied genome-wide transcript profiling in various longevity and aging models, e.g., in calorically restricted *Drosophila* flies [76]. It was repeatedly documented that calorie restriction extends lifespan in various model organisms [77]. In this context, Rogina and Helfand reported that the transcription factor Sir2 is centrally involved in the lifespan-extending molecular pathway in *Drosophila*. An increase in *Drosophila* Sir2 prolonged lifespan, whereas a decrease in dSir2 inhibited the lifespan-extending properties of calorie restriction [78].

### 2.6. Spontaneous and Induced Locomotor Activity

The spontaneous and induced locomotor activity of flies can be measured to assess fitness, locomotor ability and circadian rhythm.

Already in 1967 Seymour Benzer invented a light countercurrent assay to study locomotor activity and behavioral responses of fruit flies [79]. In a follow-up study, clock genes were discovered in *Drosophila*, which unraveled important molecular targets of the circadian network [80]. 

**Figure 8:**
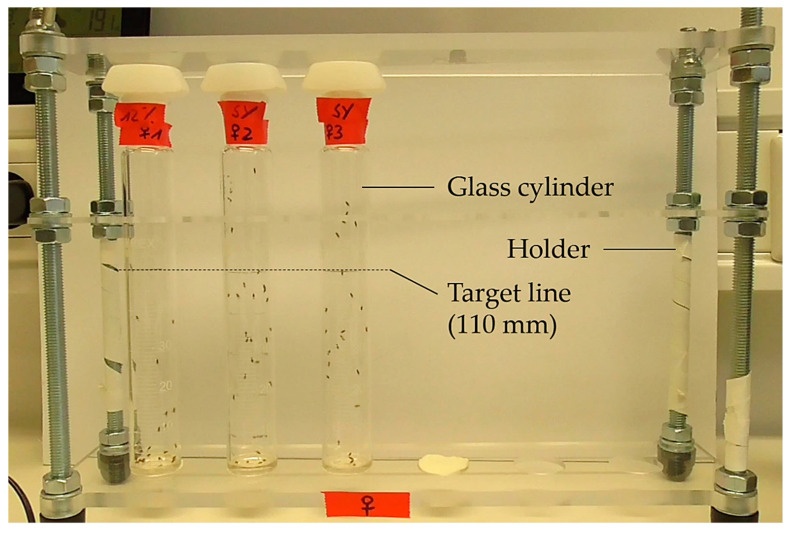
Rapid iterative negative geotaxis (RING) assay. A defined number of flies are inserted into empty vials or cylinders with a target line (marked here at 110 mm). The vials or cylinders are fixed in a holder (RING apparatus). After a short adaptation time, the RING apparatus is tapped on the table until all flies are on the bottom of the vials, and a photograph capturing all vials is taken. The process of tapping and photographing is repeated 10 times at 1-min intervals. Alternatively, a video of the climbing process is recorded. These photographs or video recordings are used to determine the climbing ability of the flies (modified from [83]).

The rapid iterative negative geotaxis (RING) assay, also called the climbing assay, is used for the determination of induced activity (Figure 8). Flies are inserted into vials that are fixed in a holder with a marked target line (RING apparatus). Tapping the RING apparatus makes the flies drop to the bottom of the vials. Negative geotaxis causes flies to have a natural drive to move upward as fast as possible, and the response is more rapid in younger and healthier flies than in older and/or more conspicuously disabled flies [81,82]. Combining negative geotaxis with the video recording of flies enables the identification of smaller differences in locomotor ability [83].

The recording of the spontaneous locomotor activity of *D. melanogaster* enables the analysis of locomotor and sleep patterns and provides data for circadian rhythm assessment. Common techniques are based on video recording or infrared light sensing [84].

Video recording is typically performed in homemade [84,85,86,87] or commercially available exposure chambers or arenas. These methods are comparatively inexpensive and allow individual adjustments. Additionally, the recording of individual movement patterns of the fly is possible, and minor defects in the fly’s movement can be captured. Recording is restricted by the memory capacity of the recording device [88].

**Figure 9:**
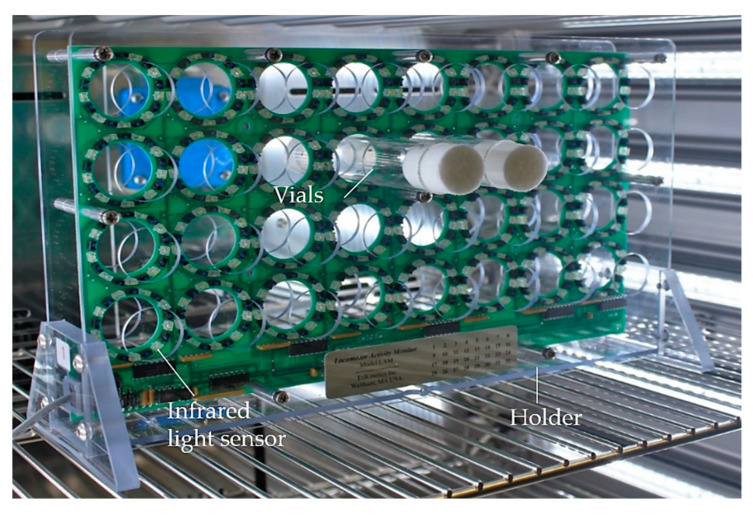
*Drosophila* Activity Monitoring (DAM) system (TriKinetics). Flies are placed in glass vials that contain experimental food and are covered with plugs. The vials are inserted horizontally into the slots of the holder of the activity monitor. In the center of each slot, an infrared light sensor is installed, which generates a signal whenever the sensor is interrupted by a crossing fly. The activity monitor is linked to a computer, and via the DAM system software, locomotion data from up to 32 vials can be recorded simultaneously (modified from [89]).

The Drosophila Activity Monitoring (DAM) system (TriKinetics, Figure 9) is based on interruption of an infrared beam [89]. Flies are placed individually or in groups in monitor vials with a continuous food supply. An infrared light sensor is installed centrally in the activity monitor, which generates a signal whenever the sensor is interrupted. The computer-based DAM system provides a simultaneous and continuous recording of the locomotor activity of several flies over several days under standard conditions. However, movement deficits are not detectable in detail, and differentiation among immobility, occupation on the medium and sleep is not possible. Moreover, the locomotion of flies with mobility deficiencies that are unable to cross the light sensor will remain undetected [88].

### 2.7. Heart Rate Measurement

The beating heart of fruit flies can be monitored by using video microscopy. Initially, flies are anesthetized, fixed, oxygenated saline is added, and surrounding organs are extracted so that the *Drosophila* heart is exposed. The investigation of parameters such as the heart rate, contractility or cardiac rhythm are measurable [22,90].

### 2.8. Fecundity, Fertility and Development as Parameters of Reproduction

**Figure 10:**
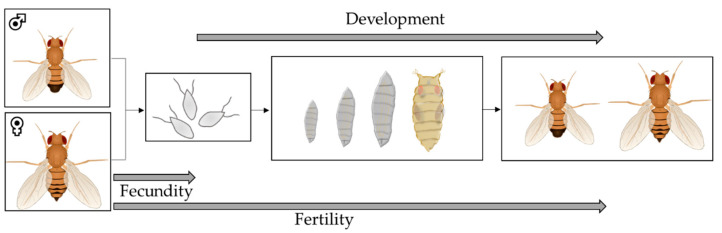
Determination of reproductive parameters in *Drosophila melanogaster*. After prefeeding males, females or both, single females, a couple or a group of flies are combined for mating. Subsequently, the reproductive performance of the female flies is examined. To determine the fecundity of flies, the eggs laid per time interval are counted. To assess fertility, the number of living offspring (larvae, pupae or adults) that develop from the laid eggs is calculated as a percentage value. Concerning the analysis of the development of the F1 generation, which considers the process from the laid egg through the individual developmental stages to the adult fly, parameters such as the development time or the sex ratio of the offspring are determined (modified from [94]).

In *D. melanogaster*, the period of time from maturation to fertility is sex-dependent. In males, the sexual maturation period length is approximately two days [91], and in females, it is about three days post hatch [92]. When females are in regular contact with males, the maximum peak of oviposition is attained after one week, after which the egg numbers continuously decline until infertility of the females occurs after approximately 50 days [93]. The important parameters for the characterization of reproduction are fecundity, fertility and development (Figure 10).

Fecundity is defined as the biological capacity for reproduction and is determined in *D. melanogaster* via the assessment of egg laying in a defined time interval [94]. In nutrition studies, virgin males and females are prefed with a test medium, and then transferred to standard medium for mating [95,96,97]. To improve egg counting, the medium with laid eggs can be stained with dyes such as Brilliant Blue FCF or rinsed and filtered [98,99]. It is also possible to assess single flies for individual fecundity [100].

Fertility describes the proportion of living offspring from laid eggs [94]. Fertility assessment involves monitoring the development of laid eggs and the documentation of the percentages of larvae, pupae and/or adult flies [95,97,101]. The influences of nutritional factors on male and female fecundity and fertility can be determined by different prefeeding and crossbreeding experiments. The effect of nutrition on male fertility, for example, can be determined by prefeeding virgin males with a standard or test medium, subsequently mating them with virgin females prefed with a standard medium and counting the viable offspring [102].

Another aspect of reproduction is the development of the offspring. The whole developmental process from eggs to larvae, pupae and adult flies or partial stages of this process can be considered. Parameters such as the development time, sex ratio, body weight, body composition and fitness of the offspring can be assessed. The consideration of several generations as a long-term approach is also an option [103,104,105].

### 2.9. Stress Assays

**Figure 11:**
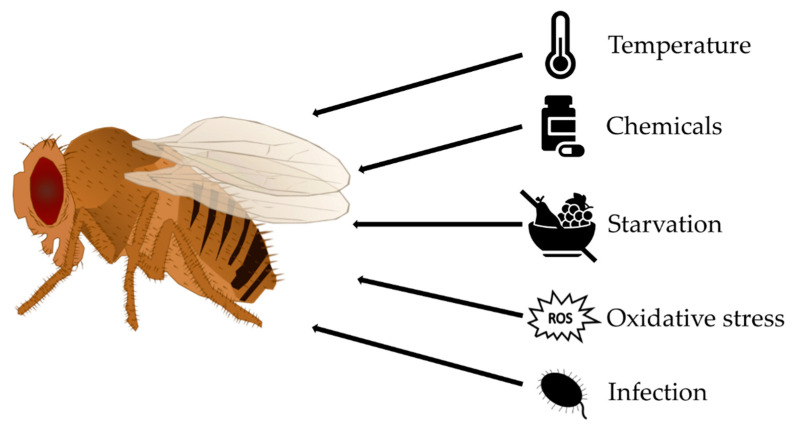
Overview of stress assays established in *Drosophila melanogaster*. The fruit fly model offers an opportunity to study the impact of dietary interventions such as high-sugar and high-fat diets on the stress tolerance of an organism. To this end, various stress assays were established, in which flies are exposed to high or low temperatures, chemicals, pharmaceuticals, starvation, desiccation or infection with pathogenic microorganisms such as *Pseudomonas aeruginosa* or *Pseudomonas entomophila*.

Environmental modifications such as extreme temperature, low food supply, infections or altered oxygen and salt concentrations act as stressors for *D. melanogaster* and may result in homeostatic imbalance [106]. The impacts of different stressors on the health and fitness parameters of *D. melanogaster* are investigated in laboratory settings (Figure 11), where stress exposure is combined with an additional method for phenotyping, such as determination of the survival rate, climbing activity, fecundity or metabolic analyses. Again, it is important to work with groups of flies of the same sex and age. To study the impact of food on stress tolerance, flies are either prefed with or maintained on experimental diets.

Thermal environment shifts such as extreme heat or cold are physical stressors for ectothermic *D. melanogaster* [107]. Numerous temperature stress protocols were developed, including short-term exposure (minutes or hours) to lethal or sublethal conditions (the latter termed hardening) as well as long-term exposure (days or weeks) to conditions within the normal viable temperature range of the fruit fly (termed acclimation). Changes in temperature can be applied as ramping temperature stress or sudden stress. A common assay is the heat shock experiment, in which larvae or adults are enclosed in a waterproof vial and placed in a water bath at an elevated temperature (commonly 38–39 °C) for a selected time. Immediately following heat exposure, the percentage of flies that exhibit heat-induced paralysis can be determined. Alternatively, after a recovery period, lethality, fecundity, fitness and metabolic function can be investigated [92,108,109].

This experimental setup of a water or ice bath can also be utilized for the opposite thermal conditions of low temperatures [110,111,112].

Furthermore, the effects of chemical stressors such as pharmaceuticals or high concentrations of heavy metals, such as copper, lead or cadmium, can be studied in *D. melanogaster*. Heavy metals and other chemical stressors are mainly applied orally as supplements of the diet. The investigation of the impacts of chemical stressors on survival, fertility, heredity and the development of offspring is an example of an application [113,114]. Elevated salt concentrations in the medium elicit osmotic or ionic stress [106].

The influences of starvation or desiccation on *D. melanogaster* can also be studied. For a starvation assay, larvae or adult flies are transferred to vials containing a liquid source such as an agar plate, wet filter paper or fluid in capillaries or a cup on the bottom [115,116]. For a combined starvation and desiccation assay, flies are placed in an empty vial with the optional addition of a desiccant [106,117]. Usually, survival rates over time (similar to the case in lifespan assays) or metabolic adaptations are determined.

In addition, *D. melanogaster* is a valuable model organism with which to study tolerance of oxidative stress. Paraquat and hydrogen peroxide are commonly employed for stress treatments. These agents are mixed into the standard medium or applied in a sucrose solution on filter paper [118,119,120]. The survival rate of flies is monitored over time or determined after a defined period of time.

To study the immune response and tolerance of pathogens, *D. melanogaster* can be challenged with several bacteria, such as *Pseudomonas aeruginosa, Pseudomonas entomophila*, or *Serratia marcescens*; viruses, such *Drosophila C virus*; or fungi, such *Aspergillus* or *Fusarium oxysporum* [121,122,123].

## 3. Obesity Phenotype of *D. melanogaster* Following a High-Sugar or High-Fat Diet

HSD or HFD feeding induces an obese phenotype with increased fat storage in *D. melanogaster* [22,124]. In addition, it was demonstrated that the hormones of energy metabolism (e.g., insulin-like peptides (*Dilps*) (a functional homolog of vertebrate insulin), unpaired2 (a functional homolog of vertebrate leptin), adipokinetic hormone (a functional homolog of vertebrate glucagon)) respond to these dietary interventions in a manner such that obesity comorbidities, e.g., insulin resistance, can be provoked [125]. Accordingly, the fruit fly has become an attractive model for studying the key metabolic processes and phenotypes of obesity and potential comorbidities. Both HSDs and HFDs are characterized by a high energy density and exhibit a low saturation effect and a good sensory quality [125].

In this context, it is of note that the terms “HSD” and “HFD” are generally not defined in terms of the quality and quantity of the added sugar or fat, which complicates the comparison of dietary treatments between studies [4].

### 3.1. High-Sugar Diet

Compared to a standard medium with a usual sucrose content of 5% (*w*/*v*), an HSD contains an elevated amount of sucrose, glucose or fructose, often with a total sugar content of approximately 20–30% (*w*/*v*) [126]. Based on the geometry of nutrition, this results in proportionally lower levels of proteins and fats [127].

Numerous studies have shown that HSD feeding leads to increased glucose, trehalose, glycogen and TAG levels [75,128,129] and decreased protein levels [75] (for details, see Table 3). Furthermore, HSD feeding leads to elevated food intake [75], impairment of carbohydrate homeostasis [130] and insulin resistance [129]. Overall, impacts of single components, such as sugar and yeast, in the medium, as well as the total energy intake, are evident in the phenotypes [75].

The HSD-induced increase in food intake and TAG storage is stimulated by the alteration of satiety behavior and disruption of sweetness detection. An HSD stimulates the hexosamine biosynthesis pathway, causing an accumulation of O-linked *N*-acetylglucosamine (O-GlcNAc) in the sweet taste neurons, which results in an increased activity of O-GlcNAc transferase, and consequently, a decreased neuronal activity [131]. Additionally, the consumption of an HSD following a fasting period leads to a modification of nutrient utilization, for example, by increasing the activity of the tricarboxylic acid (TCA) cycle [132].

Furthermore, in many publications, it was demonstrated that stress resistance is diminished after the ingestion of an HSD. Extreme cold leads to increased post-cold mortality and extended chill coma recovery time. The sugar concentration and the type of sugar (sucrose, fructose, glucose, trehalose) was observed to influence survival after cold stress [130]. Additionally, the activation of the innate immune system via the Toll and c-Jun *N*-terminal kinase (JNK) signaling pathways could also be induced following ingestion of an HSD [133].

An HSD results in detrimental changes in health and fitness parameters, such as reductions in flying and climbing ability [134] and survivorship [75,135]. Even HSD exposure at a young age induces a long-term decline in lifespan [136].

In this manner, the metabolic effects of an HSD emerge independently of hydration, while the shortening of lifespan following HSD consumption may be reversed by water supplementation. Thus, a shortening of lifespan may not be directly induced by the metabolic consequences of HSD consumption [137].

Alterations at the tissue level are also caused by HSDs. In heart tissue, the dysregulation of the hexosamine biosynthetic pathway leads to increases in hexosamine flux and heart defects, characterized by irregular beating patterns and reduced cardiac contractility [135].

Furthermore, an HSD alters the intestinal tissue, which is associated with the dysfunction of intestinal cell membranes, changes in bacterial composition [138], increased intestinal permeability and the disruption and disorganization of actin filaments in the midgut [139]. These alterations are attributed to the elevated differentiation of intestinal stem cells resulting from the upregulation of the c-Jun *N*-terminal kinase signaling pathway, downregulation of the Janus kinase/signal transducer and activator of transcription signaling pathway [138].

In addition, an HSD is associated with the morphological and functional alteration of the Malpighian tubules, characterized by cytoskeletal deformities, enhanced oxidative stress, apoptosis [140], increased deposition of uric acid stones in the Malpighian tubules and hindgut and reduced tubule secretion rates [137]. This state is linked to interference with purine biosynthesis, resulting in enhanced uric acid production, the generation of a dehydration state and acidification of the tubules and the intestine [137].

Moreover, an HSD causes neurodegenerative modifications of the eyes, e.g., increased disorganization of ommatidia, enhanced apoptosis and negative alterations in autophagy, which are partially caused by oxidative stress via ROS. Consequently, deficits in light sensitivity and visual disturbances are increasingly evident [141].

In addition, several studies indicated that decreases in reproductive capacity [142] and genetic programming [128,143] are consequences of HSD consumption. Overnutrition of the maternal F0 generation affects the predisposition for the development of an overweight phenotype under conditions of high food supply in the offspring. L3 male larvae of the F1 and F2 generations have increased whole-body glucose and trehalose levels, while females of the F2 generation exhibit increased trehalose levels and decreased TAG levels. Modifications including gene expression alterations suggest that changes in carbohydrate homeostasis and nutrient storage occur in subsequent generations [128].

In males, HSD feeding modifies genetic programming, which influences the metabolism of the subsequent F1 generation. Males of the F1 generation are predisposed to an obesity phenotype and exhibit increased body weight and TAG storage when fed an HSD compared to a normal diet. Additionally, the adipose area and lipid droplet sizes are enhanced, food intake is increased, and the starvation sensitivity of males of the F1 generation is elevated. However, no effect of the paternal diet on the development time, offspring quantity or sex ratio is observed [143].

### 3.2. High-Fat Diet

Similar to HSDs, HFDs can also be applied to generate an obesity phenotype of *D. melanogaster*. To create an HFD, an additional fat source, such as 20–30% (*w*/*v*) coconut oil or 15% (*w*/*v*) lard, is added to the normally low-fat standard medium [126].

Numerous studies demonstrated that an HFD causes increased TAG, glucose, and trehalose levels and alterations in glucose and insulin homeostasis [8,22,110,134] (for details, see Table 3). An HFD was shown to have a more severe impact than an HSD [134]. However, an assessment of the mating state of females indicates an interaction with the HFD-induced alteration in body composition, as effects are not observed in virgin females [144].

An HFD contributes to obesity via metabolic changes accompanied by modifications of sensory cognition and the perception of hunger and satiety [145,146]. Sensory cognition is mediated by a reduction in *DmOrco* expression in the peripheral olfactory organ and modification of gene expression in antennae, e.g., with regard to the olfactory receptors and insulin signaling pathway [146]. Additionally, an HFD provokes modifications in the central nervous system, such as the suppression of neuronal autophagy in octopaminergic neurons, resulting in the modification of hunger and satiety perceptions [147].

Furthermore, an HFD adversely affects stress resistance, e.g., resistance to exposure to extreme cold and anoxia [110]. The increased stress susceptibility and reduced health status lead to detrimental impacts on locomotor activity, flight ability and survivorship [8,110,134,144,148].

HFD-induced alterations in tissues can be observed. HFD consumption leads to the upregulation of phospho-protein kinase B (Akt) levels, which causes the inhibition of the transcription factor, Forkhead Box O (*dFOXO*). The downregulation of nuclear gene expression and increased activity of the *Drosophila* target of rapamycin (dTOR) follows, which in turn inhibits spargel and activates FA synthases (FASs). These metabolic adaptations ultimately promote fat accumulation, lipotoxic heart dysfunction and change skeletal muscle physiology (e.g., causing the disorganization of actin-containing myofibrils) in *D. melanogaster* [22,23,134].

Moreover, it was shown in many studies that the reproductive capacity (e.g., fecundity) of females is decreased [144], which is affected by the deterioration of activity and flight capacity and changes in the visual (body size) and nonvisual (pheromones) sex characteristics of female flies [149].

## 4. Molecular Changes Following a High-Sugar Diet and High-Fat Diet

*D. melanogaster* phenotyping in response to dietary factors such as HSD and HFD can be complemented with transcriptomic and metabolomic analyses to unravel the underlying molecular mechanisms, as indicated below.

### 4.1. Changes in Transcriptome

In recent years, several studies were published that evaluated the relation between the intake of either an HSD or HFD and gene expression patterns in *D. melanogaster*. An overview summarizing the different dietary regiments and the main outcomes including the number and examples of up- and downregulated genes is given in Table 4.

#### 4.1.1. Transcriptional Changes Induced by HSD

HSD consumption considerably affects the development of fruit flies, especially at larval stages. Palanker Musselman et al. (2011) analyzed whole-body gene expression in male *Canton-S* larvae, aiming to identify differences in gene expression patterns following the ingestion of either a control diet or an HSD containing 1 M (equivalent to 34%) sucrose for a short-term (for 12 h, starting at mid-L3 stage) or long-term (egg to wandering L3 stage) period. Both short-term and long-term HSD intake increased the mRNA levels of genes involved in glucose transport, FA and trehalose synthesis and TAG storage. Short-term HSD ingestion increased the transcription of genes that regulate glycogenolysis and the pentose phosphate shunt and reduced the expression of genes involved in glycolysis. Long-term HSD feeding elevated the expression of genes that regulate gluconeogenesis and β-oxidation. In this regard, marked alterations in the transcription of *dFOXO* downstream target genes were evident. These findings indicate causes for the development of an insulin resistance phenotype in L3 larvae following HSD treatment [129].

In contrast, a short-term feeding (24 h) with an HSD containing only 16% sucrose did not alter gene expression patterns of L3 larvae from two iso female wild-type strains (*Iso-SD1* and *Iso-SD3*). The authors conclude that *D. melanogaster* are fruit breeders and adapt much better to sugar-rich nutrition than other *Drosophila* species (e.g., *D. arizonae*, *D. mojavensis*) that are cactus breeders; therefore, no changes at the transcriptional level are observed following HSD consumption [150].

Starting from eggs, Loreto et al. (2021) reared *Oregon-R* flies on a 30% sucrose HSD and 2.5% sucrose control medium, respectively. However, in this study, whole-body gene expression was not investigated in larvae but in 7-day old adults. HSD-fed *D. melanogaster* exhibit an up-regulation of genes involved in ribosomal biogenesis and a down-regulation of genes involved in energy metabolism, including glycolysis, TCA cycle and ATP synthesis, as well as genes involved in insect development. Together with the downregulated expression of *Actin* (*Act88F*) these changes in gene expression may result in the disruption of muscle development and mitochondrial dysfunction, which is phenotypically evident in developmental delay and muscle atrophy. In addition, the expression profile of flies fed with 30% HSD, in terms of the insulin-like pathway, indicates that flies may develop type 2 diabetes mellitus-like phenotype [151].

Hemphill et al. (2018) fed an HSD (20% sucrose) for 7 days to *Oregon R-C* females and examined gene expression in the fly heads. In good accordance with the increased energy intake, genes that function in peptide and carbohydrate metabolism (protein cleavage, enzyme activation, and macromolecule processing) were found to be enriched, predominately including genes involved in sugar/carbohydrate processing. Furthermore, genes relating to checkpoint kinases were upregulated by the HSD, which together with the decreased expression of genes related to the cell cycle, was discussed in the context of high-dietary-sugar-induced DNA damage. The upregulation of the transcription of heat shock proteins, genes involved in antimicrobial defense and genes of apoptotic processes, was also evident, indicating that HSD-fed animals experienced stress. In good accordance with the increased TAG storage, *Akh* is downregulated and *heimdall* is upregulated by the HSD [152].

As described by Ng’oma et al. (2020), 10 days of feeding of an HSD containing 1 M sucrose alters multiple genetic networks (examined with RNA-seq) in outbred females (*Drosophila* Synthetic Population Resource) in comparison to control-fed flies. Although genes encoding the proteins of insulin/insulin-like signaling/the TOR pathway, including *Dilp5*, *Rheb*, and *Sirt2*, showed a significant elevation in expression, the effect of an HSD was not limited to key genes but rather affected a wide range of biological pathways and cofactors [153].

**Figure 12:**
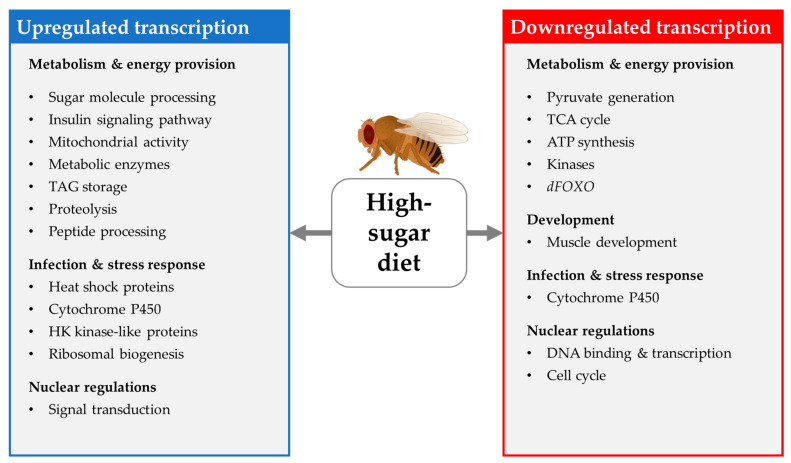
Transcriptional alterations following intake of a high-sugar diet (HSD) in *Drosophila melanogaster*. Ingestion of an HSD causes upregulation (blue box) and downregulation (red box) of genes involved in metabolism and energy provision, infection and stress responses, nuclear regulation, and development (modified from [129,136,151,152,153]).

An HSD that is solely administered in early adult life affects long-term health outcomes at older ages. The reversible inhibition of the activity of the transcription factor *dFOXO*, after a week-long treatment with an HSD containing 40% sucrose, was indicated by RNA-seq analyses. The inhibition of *dFOXO* alters the downstream gene transcription of chromatin/nucleosome remodelers and histone-modifying enzymes in wild-type female flies. Moreover, the inhibition of dFOXO leads to reprogramming at the transcriptome level, which affects lifespan [136]. This summary of transcriptome studies indicates that different dietary treatment regiments (varying sugar contents of the HSD and periods of intervention) targeting different sexes, wild-type strains and stages of the fly life cycle (larvae feeding only, larvae to adult feeding, adult feeding only) were applied to examine the impact of HSD on gene expression. In addition, gene expression was analyzed in different parts of the fly body (whole body, heads only). Although this heterogeneity hampers data comparison, we tried to extract general trends in HSD-induced gene expression, which are summarized in Figure 12.

#### 4.1.2. Transcriptional Changes Induced by HFD

Ruden and co-workers [154,155] presented the combination of high dimensional biological data with complex diseases and quantitative traits and gave guidelines for the design, analysis and interpretation of corresponding experiments. They further released important work in the field of nutrigenomics (as the study of gene-nutrient interaction) by using *Drosophila* “whole genome” arrays in response to various dietary conditions.

In all studies that will be presented below, the HFD comprises 20% (*w*/*v*) coconut oil and was fed for 7 days to adult fruit flies.

The impact of consumption of an HFD on the transcriptome was investigated in female *w^1118^ D. melanogaster* by Heinrichsen et al. (2014). In particular, the immune response, carbohydrate binding, iron transport and extracellular secretion are modified by the altered mRNA levels. In addition, the expression levels of genes that are associated with the metabolism of amino acids and glycerophospholipids, as well as the degradation of sugars and waste metabolites, are changed. Another factor is the diminishment of *CG9510* expression (the homolog of mammalian *ASL*), which is involved in carbon and nitrogen metabolism. Downregulation of *CG9510* expression unfavorably increases fat storage, impairs cardiac function, decreases tolerance to stress and shortens lifespan [156].

Ingestion of an HFD in adult *w^1118^* female *D. melanogaster* affected genes expressed in heads encoding odorant/pheromone proteins, cytochrome P450, monooxygenase, and enzymes that account for antioxidant capacity [157]. The increased transcription of odorant binding proteins may be associated with efforts to search for other nutrient sources. Genes that encode enzymes that are related to nucleotide metabolism are also transcribed to a greater extent in the head of HFD-fed flies than in controls. HFD mainly downregulates the expression of genes that encode factors that are involved in DNA replication, mitosis, cell cycle regulation, RNA binding and neurogenesis. Downregulation of the neuronal expression of these genes was discussed in the context of a higher response to oxidative stress, neuronal substrate exhaustion and HFD-induced mild neuronal deterioration and impairment of short- and long-term memory as well as disturbance of neurophysiological mechanisms (e.g., neuromuscular signaling) and locomotion. In addition, ingestion of an HFD is associated with a reduction in the expression of doppelganger of brummer (*dob*), which is related to an increase in fat storage [157].

However, the previously reported impact of the expression of *dob* on fat accumulation cannot be validated, in that a lack of *dob* activity in adult *D. melanogaster* mutants has not been found to modify the TAG content. It can be inferred that *dob* exhibits *bmm* activity in the fat body but remains nonessential for the regulation of chronic TAG storage [158].

Stobdan et al. (2019) reported that feeding with an HFD induces shifts in transcription patterns in adult *w^1118^ D. melanogaster* compared to the respective controls that are sex and organ-specific (measured via RNA-seq of heads and bodies) [159]. Males are affected to a greater extent than females, as the transcription of a higher number of genes is altered in males (Table 4). In males, downregulation of whole-body glycoside hydrolases (e.g., lysosomal α-mannosidase (*LManIII–VI*)), which are significantly involved in carbohydrate metabolism, at the transcriptional level is evident. The expression of these genes may be related to sperm quality and function in *D. melanogaster* [160]. In addition, the expression of genes encoding proteins that are associated with stress and the immune response is altered in the heads of males. This may alter the stress tolerance of males following HFD consumption [161]. In females, alteration at the transcriptional level is present for genes involved in lipid metabolism. Ingestion of an HFD induces transcription of numerous related genes in the heads of flies. In males and females, the transcription of Cytochrome P450 4e3 (*Cyp4e3*), which is associated with antioxidant capacity, and takeout (*to*), which is associated with hyperphagic behavior of the fruit fly, is upregulated. In the fat body, the changes in the expression profile likely contribute to the regulation of lipid homeostasis in both males and females [159].

Results published by Azuma et al. (2019) validate the abovementioned findings, as the researchers found that consumption of an HFD similarly modified whole-body gene expression (determined by RNA-seq) in adult *Canton-S D. melanogaster*. Genes involved in the regulation of the energy metabolism, fatty acid synthesis and modification as well as in carbohydrate metabolism were affected. Accordingly, changes in the transcript frequency of these genes were associated with alterations in glucose metabolism and gluconeogenesis. Again, the transcription of genes that encode key factors in the oxidative stress response was increased, while genes encoding antibacterial proteins was diminished, leading to impairment of immune system function. In females, the expression of genes that are critically involved in egg production, was decreased, impairing oogenesis [162].

Hemphill et al. (2018) examined the HFD-induced changes in gene expression in the heads of *Oregon R-C* females. Similar to HSD investigated in the same study, ingestion of the HFD upregulates the expression of genes encoding proteins that are involved in peptide and carbohydrate metabolism. Upregulation of transcription of heat shock proteins, indicating induction of the stress response, and of genes of antimicrobial peptides and immune function and the apoptotic processes was also evident. On the other hand, intake of an obesogenic diet leads to downregulation, e.g., of the expression of *heimdall* (*CG4500*), which affects TAG storage and disturbs sleep homeostasis [152]. In addition, the transcription of genes related to chitin metabolic processing and metabolic functions is increased, which may promote circulation of excess metabolites. Nutrient storage, signal transmission and pyridoxal phosphate function are downregulated at the transcriptional level by an HFD.

**Figure 13:**
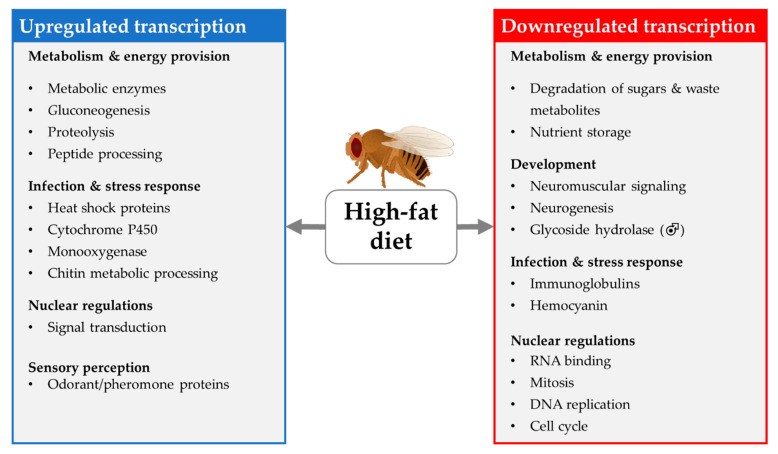
Transcriptional changes in *Drosophila melanogaster* resulting from high-fat diet (HFD) feeding. An HFD induces upregulation (blue box) and downregulation (red box) of genes involved in metabolism and energy provision, infection and stress responses, nuclear regulation, sensory perception, and development (modified from [152,156,157,159,162]).

Compared to the HSD studies presented above, the studies that examined the changes in the transcriptome of fruit flies in response to an HFD are by far more homogeneous in terms of the dietary regiment. In most cases, 20% coconut oil was fed for 7 days to adult flies. Accordingly, the data lead to a more coherent picture of the HFD induced transcriptional changes that summarized in Figure 13. On the other hand, several open questions regarding, e.g., the effects of fat quality and fat quantity on differential gene expression remain to be elucidated.

### 4.2. Changes in Metabolome

Only a few studies focusing on the effect of HSD or HFD intake in larvae or adult *D. melanogaster* on metabolomics were published in recent years.

Both the genotype (16%) and the diet (9%) considerably affect the metabolome of *D. melanogaster*, whereas the interaction of these two factors (2%) apparently exerts minor effects on the variance of the fruit fly metabolome [163,164]. The presumed impacts of the ingestion of an HSD and HFD on *D. melanogaster* was investigated by metabolomics analyses, including information on the dietary intervention protocols and examples of up- and downregulated metabolites. An overview is presented in Table 5.

#### 4.2.1. HSD Metabolome

Colinet et al. (2013) performed a feeding study with wild-type *D. melanogaster* (a mixture of two wild populations collected in Brittany, France), which were consecutively administered diets containing sucrose, glucose, fructose and trehalose at concentrations between 0 and 1000 mM from the larval to adult stages, and conducted metabolic fingerprinting by GC/MS 5 days post eclosion [130]. The sucrose- and fructose-containing diets modified, in particular, contain partially overlapping sets of metabolites of the carbohydrate metabolism. The elevated malate concentration, following the application of a sucrose- and fructose-containing diet, was suggested to be attributable for an altered lipid biosynthesis and a dose-dependently-increased fat accumulation. It was found that all sugar-supplemented diets were absorbed by the fruit flies and accumulated as fat. In addition, the intake of a glucose- and trehalose-enriched diet affected carbohydrate and amino acid metabolism. GABA concentrations dose-dependently decreased following the short-term ingestion of a glucose- or trehalose-supplemented diet. Low GABA concentrations affected the production and release of Dilps in insulin-producing cells [165]. Overall, a concentration-dependent increase in sorbitol level was detectable following ingestion of either diet, which resulted from activation of the polyol pathway due to the presence of high concentrations of fructose and glucose. Additionally, increased ROS production arose from modifications of the polyol pathway [130].

Simard et al. (2020) investigated the impact of the 15-day ingestion of an HSD containing 18% and 30% sucrose on the metabolite profiles of adult wild-type *w^1118^* flies and flies with mild mitochondrial pyruvate carrier (MPC) 1 deficiency using ^1^H NMR spectroscopy [166]. The modification of a low number of metabolites, e.g., increases in fructose, glucose-6-phosphate and proline levels, as well as slight decreases in leucine, malate and citrate levels, was detected in *w^1118^* flies following the ingestion of the HSD. In contrast, flies with mild MPC1 deficiency exhibited an accumulation of numerous metabolites, such as sugars (e.g., glycogen, fructose, glucose), glycolysis intermediates (e.g., glucose-6-phosphate) and amino acids (e.g., glycine, proline, β-alanine). MPC is nested in the inner mitochondrial membrane and is essential for pyruvate transport into the mitochondria [167]. Sugar accumulation and the diminishment of pyruvate-stimulating respiration indicated metabolic inflexibility in MPC1-deficient flies, leading to a depletion of homeostasis [166].

Gillette et al. (2020) investigated the interaction of genes and diet in *w^1118^* and split ends (Spen)-depleted *D. melanogaster* larvae following treatment with an HSD (1 M corn syrup) via ultra-high-performance LC/MS. Spen, an extremely large, RNA-binding protein, is a key regulator of the energy balance in the fruit fly. Its depletion led to defects in energy metabolism (e.g., β-oxidation) and increases in TAG storage. In *w^1118^* flies, the total metabolic difference caused by the ingestion of an HSD amounted to a total of only 3.6% compared to the levels in controls. In general, the administration of an HSD led to a reduction in strain-dependent differences between Spen-depleted and *w^1118^* larvae. The similar metabolic profiles in both genotypes indicated that the HSD eradicated the genetic influence of Spen deficiency on metabolism [168].

Tuthill et al. (2020) analyzed the lipid profiles in the fat bodies, hemolymph and hearts of adult *w^1118^ D. melanogaster*, following the ingestion of an HSD containing 1 M sucrose, for three to five weeks by ultra-high performance liquid chromatography—MS/MS. The relative levels of diglycerides and TAG were increased in all analyzed tissues, with TAG being the most affected by the ingestion of an HSD. In addition, the lengths of substituent chains were modified following the intake of an HSD, resulting in fewer odd-chain, esterified FAs in all examined tissues. The lower odd-chain FA content in HFD-fed fruit flies was discussed in the context of a concurrent change in the intestinal microbiome. Since odd-chain FA are generated by enteric bacteria, increased even:odd chain ratios may reflect a reduced lipogenesis by enteric bacteria. In the fat body, in general, the levels of 10 lipids increased, while those of 110 lipids decreased. The highest variability in lipid content was evident, with increases in saturated FA and monounsaturated FA substituents and a decrease in the relative proportion of double bonds. In the hemolymph, in total, the proportions of 33 lipids increased, while those of most of the lipids (131 lipids) decreased. The proportions of TAG and ether lipids changed to a smaller extent in the hemolymph than in the fat body. In addition, reductions in ceramides and sphingomyelin were evident in the hemolymph. Similarly, the alterations in cardiac tissues reflected the trends in the other tissues. In total, 44 lipids were altered in the cardiac tissue in response to the intake of an HSD [169].

Since the storage capacity of the fat body of *D. melanogaster* is limited, a surplus of FA/lipids elicits lipotoxicity in other tissues [170].

**Figure 14:**
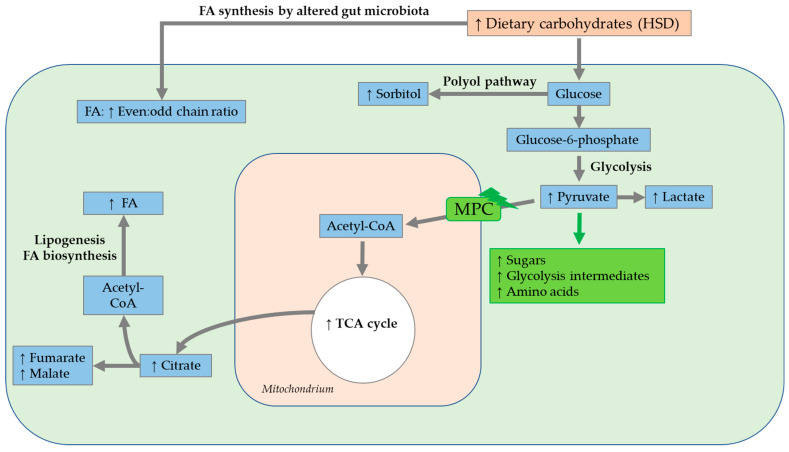
Metabolomic changes in *Drosophila melanogaster* in response to high-sugar diet (HSD) feeding. Flies fed an HSD supplemented with sucrose show elevated levels of pyruvate and lactate, which can be explained by stimulation of the glycolysis pathway. Moreover, the levels of fatty acids (FA) and stored lipids are increased, indicative of enhanced lipogenesis and FA biosynthesis [130,169]. Additionally, flies fed an HSD exhibit fewer odd-chain esterified FAs in all examined tissues, which is most likely attributable to an altered composition of the gut microbiota [169]. Ingestion of an HSD leads to increased levels of sorbitol produced via glucose utilization in the polyol pathway [130]. Effects elicited by long-term feeding on an HSD are depicted with green symbols and boxes. Long-term HSD exposure negatively affects the mitochondrial pyruvate carrier (MPC) in the inner mitochondrial membrane (lightning bolt). Mild MPC deficiency is associated with increased accumulation of sugars, glycolysis intermediates and amino acids (green box), and a reduction in pyruvate-stimulating respiration suggests metabolic inflexibility and the disruption of homeostasis [166] (modified from [130,166,169]). Acetyl-CoA, acetyl-coenzyme A; TCA, tricarboxylic acid; ↑ increase; ↓ decrease; thickness of arrows indicates flux rate.

It was also shown, in the previously mentioned study of Tuthill et al. (2020), that a long-term (3- to 5-week) intake of an HSD led to an overabundance of lipids in the fat body and heart and increased levels of palmitate in lipids, di-saturated glycerolipids and DAGEs, which were linked to lipotoxic effects. Additionally, an increase in the even:odd chain ratio following the ingestion of an HSD was evident in all tissues studied, which was again related to the alteration of the gut microbiota [169]. A summary of the outlined results following a HSD is depicted in Figure 14.

#### 4.2.2. HFD Metabolome

Oza et al. (2019) conducted untargeted metabolomics analyses (GC/MS and LC/MS) of the larval *D. melanogaster* of 16 different cultured genotypes. Larval feeding of an HFD containing 3% (*w*/*v*) coconut oil caused increased medium-chain FA and dicarboxylic FA levels in larvae, which presumably originated directly from coconut oil in the HFD. Ingestion of the HFD led to increased monohydroxy FA levels as end products of the omega oxidation pathway. Medium-chain FAs of the HFD were incorporated directly into the larval omega FA oxidation pathway as an alternative to β-FA oxidation. Additionally, larval intake of the HFD increased glucose-6-phosphate, fructose-6-phosphate and citrate levels, suggesting the involvement of glycolysis and the downstream TCA cycle. Larvae of the different cultured genotypes represented different TAG storage phenotypes with differing dipeptides, lysolipids and components of amino acid metabolism. The intake of the HFD led to increased protein catabolism or enzymatic dipeptide synthesis with increased dipeptide levels. In total, a higher network density was evident after larval ingestion of the HFD than after the intake of a control diet [171].

Heinrichsen et al. (2014) performed metabolomics analyses (GC/MS) on female *w^1118^ D. melanogaster* following the ingestion of an HFD supplemented with 20% (*w*/*v*) coconut oil for 7 days. The intake of the HFD led to increases in the total metabolic rate and oxygen consumption and decreases in fumarate, malate and α-ketoglutarate related to the TCA cycle. The proportions of total FAs, lactate, pyruvate, urea and uric acid were increased, while those of numerous amino acids were decreased, which indicated alterations in carbohydrate and amino acid metabolism. Additionally, the ingestion of the HFD increased FA oxidation in mitochondria, leading to increased levels of acetyl-coenzyme A (acetyl-CoA). As pyruvate is not primarily used for acetyl-CoA synthesis, pyruvate accumulation and conversion to lactate occurred. In parallel, amino acid oxidation was increased to provide anaplerotic substrates for the TCA cycle. This alteration in nitrogen metabolism was also evident at the transcriptional level with regard to the expression of *CG9510* (see section Transcriptomics: Application to *D. melanogaster*) [156].

Cormier et al. (2021) validated the abovementioned findings of Heinrichsen et al. (2014), since a high-substrate supply of FAs, provided by the intake of an HFD containing 20% (*w*/*v*) coconut oil for 10 days, caused an overload of the TCA cycle with increased levels of cytosolic nicotinamide-adenine dinucleotide (NADH) and acetyl-CoA in male *w^1118^* flies, leading to an inhibition of pyruvate usage and increased pyruvate accumulation. TCA cycle intermediates and amino acids replenished the TCA cycle. In total, metabolomics analyses (NMR spectroscopy) indicate an increase in TCA cycle-related metabolites, as well as an accumulation of seven metabolites associated with carbohydrate metabolism and eight amino acids following the 10-day ingestion of an HFD. The accumulation of 25 metabolites totally combined with mitochondrial dysfunctions suggests a metabolic of an inflexibility following the intake of a HFD [172].

**Figure 15:**
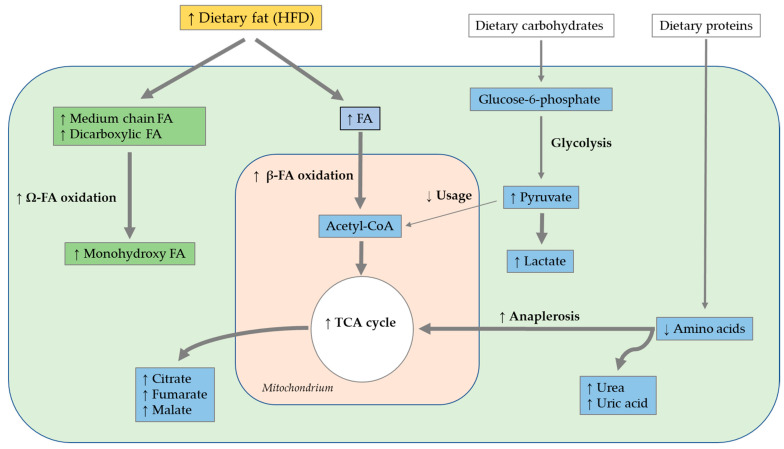
Metabolomic changes in *Drosophila melanogaster* fed a high-fat diet (HFD). Metabolites that are affected in adult flies are in blue boxes, and those that are specifically altered in larvae are in green boxes. In adult flies, an HFD led to enhanced levels of acetyl-coenzyme A (acetyl-CoA), indicating an increased β- oxidation of fatty acids (FAs). In line with this, citrate, malate and fumarate concentrations are increased, which points to the stimulation of the tricarboxylic acid (TCA) cycle, fueled by the acetyl-CoA surplus. An overload of the TCA cycle by β-oxidation-derived acetyl-CoA is further supported by elevated levels of pyruvate and lactate, indicating a decreased utilization of glycolysis products by the TCA cycle [156,172]. In addition, amino acid levels declined, and products of amino acid catabolism, such as urea and uric acid, were elevated. This points to an increased demand for amino acids to provide anaplerotic metabolites for the TCA cycle [172]. In larval *D. melanogaster* (green boxes), an increase in Ω- FA oxidation is also evident [171] (modified from [156,171,172]). ↑ increase; ↓ decrease; arrow thickness indicates flux rate.

Apart from testing an HSD (mentioned above), Gillette et al. (2020) fed *w^1118^* and split ends (Spen)-depleted *D. melanogaster* larvae with an HFD (15% (*w*/*v*) coconut oil), before conducting metabolome analyses via ultra-high-performance LC/MS. In *w^1118^* flies, the total metabolic difference caused by ingestion of the HSD amounted to a total of only 3.6% compared to the level in controls, while the difference following the intake of an HFD was comparatively high at 11.1%. The *w^1118^* flies demonstrated a higher metabolic flexibility and substantial resistance to dietary extremes such as high-fat intake, compared to the Spen-depleted flies. In general, the administration of an HSD and HFD led to a reduction in strain-dependent differences between Spen-depleted and *w^1118^* larvae. Thus, only two metabolites, acetylcholine and L-carnitine, among 140 detected metabolites differed in HFD- versus control diet-fed flies. The similar metabolic profiles in both genotypes indicated that the HSD and HFD eradicated the genetic influence of Spen deficiency on metabolism [168]. A summary of the outlined results following a HFD is shown in Figure 15.

## 5. Relevance of Findings in *D. melanogaster* for Other Species

Comprehensive phenotyping of the model organism *D. melanogaster* is feasible in the context of chronic metabolic diseases such as obesity and diabetes. To what extent findings in *D. melanogaster* may be translated to other mammalian species, such as laboratory rodents and humans, will be discussed in the section below.

### 5.1. Lipid Metabolism

The evolutionary and/or functional conservation of multiple tissue and organ structures, such as the central nervous system and the digestive tract, reveals similarities in signaling pathways and regulation of lipid metabolism between *D. melanogaster* and mammalian species.

In *D. melanogaster*, the digestion and absorption of lipids predominantly proceed in the midgut, which can be broadly subdivided into the anterior, middle and posterior midgut and by morphology into 10–14 subregions [173]. In metazoan animals, ingested macronutrients, including lipids and phospholipids, must be enzymatically degraded within the alimentary tract before they can be taken up by enterocytes. Although the intestinal digestion of dietary lipids by the fruit fly is still poorly understood, the current data indicate that crucial aspects are conserved with respect to the situation in mammals. The *Drosophila* genome encodes several putative lipases exhibiting midgut expression [174]; currently, only a single intestinal TAG lipase, Magro, has been functionally described [175,176]. Nevertheless, it is highly likely that, similar to gastric, lingual and pancreatic lipases in mammals, ingested lipids (TAGs, phospholipids) are initially hydrolyzed by different lipases, namely neutral and acidic lipases in the fruit fly. Based on studies with other insects, it can be also assumed that lipid digestion and absorption in *Drosophila* is promoted by emulsification through the generation of FA-amino acid complexes, glycolipid complexes and FA-lysophospholipid micelles [177]. However, bile salts, which represent the key emulsifiers in mammalian dietary lipid digestion, are lacking in the fruit fly. In mammals, the lipase products monoacylglycerol (MAG), DAG and free FAs form mixed micelles with lysophosphatidic acid, cholesterol, bile salts and fat-soluble vitamins capable of diffusing into enterocytes. In addition, MAG and free FAs pass the enterocyte membrane presumably by passive diffusion and active transport and are re-esterified back into TAGs. Similarly, free FAs, MAG, DAG and sterols are absorbed through the *Drosophila* midgut epithelium most likely facilitated by the emulsification and/or driven by diffusion via a concentration gradient caused by lipid re-synthesis in enterocytes. Whether transporter proteins are involved in this process has not yet been shown. A surplus of dietary lipids can result in TAG accumulation and transient storage within cytosolic lipid droplets of the enterocytes [178,179]. Additionally, FAs are produced in enterocytes via tissue-autonomous de novo lipogenesis, primarily by the enzymes acetyl-CoA-carboxylase and FA synthase, from acetyl-CoA (e.g., excess dietary carbohydrates). However, unlike in mammals, the de novo biosynthesis of cholesterol was not observed in *D. melanogaster*. Hence, the fruit fly is a cholesterol auxotroph and depends on the dietary intake of cholesterol mediated by Niemann-Pick-type C-1b [180,181]. FAs from the diet and de novo lipogenesis are converted via the glycerol-3-phosphate pathway or, alternatively, the MAG pathway to DAG.

For the interorgan transport of lipid molecules, such as FAs, DAG, TAG and sterols, from enterocytes to other tissues via the blood/lymph or hemolymph system, the presence of specific lipid-binding proteins is required. In *D. melanogaster*, DAG, cholesterol, cholesteryl ester and phospholipids are loaded onto the apoB-family lipoprotein, lipophorin, mediated by another apoB-family lipoprotein, a lipid transfer particle, before the resulting lipoprotein complex is secreted into hemolymph [181,182]. In mammals, in intracellular metabolism, TAGs combine with cholesterol and apolipoprotein to form chylomicrons, which are then released via the lymphatic system into the blood circulation. In contrast, short- and middle-chain FAs are directly bound to albumin and are transported in the blood circulation without re-esterification. The lipophilic substances in the lymphatic system and blood circulation proceed to be transported as lipoproteins, which are distinguished by different apoproteins, each intended for specific transport particles, enzymes and receptor binding in tissues. At endothelial cell membranes, TAGs in chylomicrons undergo cleavage catalyzed by lipoprotein lipase into glycerol and FAs. The FAs can be directly transferred to muscle for energy synthesis or to adipose tissue for storage. Fat storage occurs primarily by re-esterification into TAGs in white adipose tissue located both intra-abdominally and subcutaneously [183]. The chylomicron remnants that remain after hydrolysis are metabolized in the liver. In addition, under conditions of carbohydrate excess, de novo lipogenesis mainly occurs via the acetyl-CoA-carboxylase and FA synthase enzymes from acetyl-CoA in adipose tissue and liver tissue [184]. The lipophilic products from endo- and exogenous supplies are transported as very-low-density lipoproteins (VLDL) in the bloodstream and undergo cleavage catalyzed by lipoprotein lipase. After the hydrolysis of VLDL by lipoprotein lipase, intermediate-density lipoproteins remain, which are further converted into low-density lipoproteins. These mainly transport cholesterol to the cells. Excess cholesterol and phospholipids are shuttled back to the liver by high-density lipoprotein [185,186].

The accumulation of energy reserves stored as fat enables energy to be available on demand in periods of nutritional deficiency. The fat mobilization process is highly conserved among fruit flies and mammals. In mammals, lipolysis involves all tissues (e.g., brown and white adipose tissue, muscle tissue and heart tissue), with most TAG storage in white adipose tissue. In energy-deficit conditions, cyclic adenosine monophosphate binds to protein kinase A (PKA), which catalyzes the phosphorylation of hormone-sensitive lipase (HSL) and perilipin 1. The phosphorylation of perilipin 1 leads to increased recruitment of HSL at the lipid monolayer of the lipid droplets, where lipolysis is stimulated. Adipose TAG lipase (ATGL) hydrolyzes TAGs into DAG, and some FAs play an important role in β-oxidation [187,188]. DAG is then hydrolyzed by Hsl into MAG, which in turn is hydrolyzed by monoacylglycerol lipase into glycerol and FAs. The FAs can be utilized for energy synthesis, re-esterification into TAGs and the synthesis of, for example, phospholipids and cholesteryl esters [186].

In the fruit fly, energy requirements can be met by the lipolysis of stored TAGs primarily in the fat body by the lipase BRUMMER, a homolog of human ATGL. Alternatively, other TAG lipases, which are dependent on adipokinetic hormone signals, can be involved in lipolysis. Similar to mammals, lipolysis is regulated by the PAT (perilipin, ADRP, TIP47) domain proteins, PLIN1 and PLIN2. In energy-deficit conditions, PLIN1 is phosphorylated by PKA, leading to the recruitment of Hsl at the lipid monolayer of lipid droplets and the stimulation of lipolysis [187]. The resultant DAG can then be hydrolyzed into FAs, possibly by *Drosophila* Hsl, to become available for β-oxidation and synthesis [179].

### 5.2. Obesity and Related Comorbidities

Obese *Drosophila* phenotypes can be generated by either genetic or dietary means. The genetic factors influencing obesity include genetic variations such as the alteration of a specific gene (monogenic factors), multiple genes (polygenic factors) or a combination of obesity with other organic or systemic dysfunctions (syndromic factors) [189]. Based on the evolutionary conservation of genes, findings from genetic studies on *D. melanogaster* may be at least partly transferred to other species such as mammals, especially in the context of disease association. For example, the downregulation of the genes *bmm* (a homolog of *ATGL*) and *AKHR* in *D. melanogaster* was shown to disturb fat homeostasis, resulting in the inhibition of the release of stored fat, causing obesity under unchanged dietary conditions [176,190]. Similarly, investigations with laboratory rodents and human studies indicated a connection between the downregulation of the *ATGL* gene, for example, by a pharmacological ATGL inhibitor and the accumulation of TAGs in various tissues [191].

Following industrialization and modernization, a “Westernization” of lifestyle and diet has occurred in humans, characterized by a decrease in daily physical activity and intake of an energy-dense Western diet rich in processed foods, meat, dairy products and sweets [192,193]. In this regard, the daily energy intake per person among human populations worldwide has increased over the last 30–50 years [194]. A permanent excess of energy intake due to consumption of energy-dense foods, accompanied by poor physical activity, causes overweight, obesity and comorbidities in humans [195,196,197,198] and animals [199,200,201]. Similarly, the obesity phenotypes in *D. melanogaster* induced by HSD and HFD feeding are accompanied by increased fat storage [22,75,128], alterations in carbohydrate-insulin homeostasis [124,129,141], and reductions in fitness [8,22,134] and reproductive capacity [75,137,142].

The Western diet, which has a combination of high levels of fat, sugar and salt, decreases lifespan, reproduction, mitochondrial function and activity; increases TAG storage; and has stronger detrimental effects than an HSD or HFD alone. There is also evidence that the exposure of *D. melanogaster* to a Western diet causes alterations in food-seeking behavior and choice, increasing the preference of flies for a diet rich in sugar, fat and salt over the standard diet [202].

A preference for fat and sugar [203,204] and reduced taste perception [205,206] (reversible by weight loss [207]) are also found in overweight humans. However, the cause–effect relationship between these associations is mostly unclear [208].

The impact of fat quantity in the diet on the development of obesity and related diseases is widely discussed. In guidelines and recommendations in many countries, the total fat intake is considered, and reference levels of 30 percent of energy per day are set as far as human nutrition is concerned [209,210]. Overall, with probable evidence, no association between total fat intake and obesity is observed when energy adjustment is applied [211].

A study on rats and *D. melanogaster* found that a moderate HFD with 35 energy percent, compared with an isocaloric control diet without added fat, was unlikely to increase body weight and TAG storage and had a positive effect on lifespan. The isocaloric moderate HFD caused a downregulation of anabolism and upregulation of catabolism of free FAs, resulting in a downregulation of free FAs in serum and tissues. The downregulation of palmitic acid in turn upregulated the peroxisome, proliferator-activated receptor gamma (*PPARG*)/PPARG-related coactivator 1 (*PPRC1*) expression, which resulted in decreases in oxidative stress and inflammation, improving the survivorship of rats and fruit flies [212].

Life circumstances such as limited sleep, alterations in circadian rhythm (e.g., shift work), stress and intense emotions also influence eating behavior and metabolism and promote obesity.

In *D. melanogaster*, it was shown that stress induced by prolonged social isolation causes an increase in food intake and a reduction in sleep. Social isolation results in an increase in limostatin and a reduction in drosulfakinin at the transcriptional level in the head, suggesting that social isolation mimics a starvation state without a limitation of food supply [213]. Similar observations of changes in eating behavior caused by social isolation and emotional stress toward uncontrolled food intake, increased food cravings, emotional eating, binge-like eating, and, to some extent, increased restrictive eating, were obtained in laboratory rodents [214,215] and humans [216,217], predominantly in females.

Moreover, a circadian rhythm of food intake can be identified in *D. melanogaster.* Regulated by clock genes, food intake is concurrent with locomotion rhythms occurring around sunrise and sunset. Mutations in clock genes induce a shift in meal timing without affecting the overall quantity of food eaten [218]. Additionally, a shift in circadian rhythm in fruit flies causes sex- and line-dependent changes in glycogen and TAG content, with *Oregon* females demonstrating increased TAG levels [219].

Circadian rhythms of gene expression, enzyme and hormone release, transport systems, and consequently, metabolic regulation can similarly be seen in mammals. Mutations in, e.g., clock genes, also cause reductions in diurnal feeding rhythms, which can promote obesity and metabolic diseases [220,221]. For example, disturbed rhythmic insulin secretion from the pancreas predisposes mammals to diabetes [222].

In addition, it was demonstrated in laboratory rodents that an HFD provided ad libitum impaired the circadian expression of, for example, the genes, *clock* and *period circadian regulator* (*per1*), and promoted obesity. Feeding a temporary HFD in a fixed time frame (e.g., zeitgeber phase 4–8 as CT0 lights on) recovers the gene expression of clock genes and can be used for obesity prevention [221].

Diabetes mellitus can be modeled in *D. melanogaster*, with type 2 diabetes concomitant with obesity. The insulin signaling pathway in the fruit fly is evolutionarily conserved. *D. melanogaster* produces eight different Dilps, of which Dilp1, 2, 3 and 5 [223] were demonstrated to have a function homologous to that of vertebrate insulin and act in various target tissues. Dilp6 is most similar to mammalian insulin-like growth factors. In the adult fly, Dilps are produced by insulin-producing cells of the central nervous system, midgut, renal tubules, adipose tissue, ovaries and abdominal ganglion [224].

Circulating Dilps in the hemolymph bind to the *Drosophila* insulin receptor, causing the activation of chico, a homolog to adaptor protein insulin receptor substrate 1–4 in mammals. Activated chico interacts with two distinct Src homology 2 domain proteins, leading (homologous to the process in mammals) to the activation of two different cascades of the insulin signaling pathway (via extracellular-signal-regulated kinase-A and DAKT-dS6 kinase) [225,226,227,228].

The insulin signaling pathway interacts with a transcriptional feedback control system of *dTOR* and *dFOXO* with a systemic and tissue-autonomous impact on metabolic adaptation, for example, in response to varying food supply [229].

After exposure to an HSD, *D. melanogaster* demonstrates hyperglycemia, resulting in increased *Dilp2*, *3* and *5* expression [129]. The prolonged ingestion of an HSD results in decreased insulin production (reduced Dilp2, 5) and increased insulin resistance, indicating a type 2 diabetes phenotype [135,142,230]. These findings are in accordance with the results from mammals, in which hyperglycemia initially leads to increased insulin secretion and, over time, decreased insulin secretion by pancreatic β-cells [231,232].

Obesity per se and associated pathologies such as diabetes or hypertension are linked with diseases of the cardiovascular system. *D. melanogaster* and mammals exhibit many similarities in the cardiovascular system, such as a four-chambered heart structure [233], cardiac contraction-dependent transport, and similar cardiac output and flow rates in large vessels (e.g., aorta) [234], allowing, to a certain extent, for the application of the fruit fly for research on heart and circulatory diseases.

HSD and HFD intake are associated with increased cardiac fat storage, structural pathology such as collagen deposition, decreased contractility, conduction blocks, arrhythmia and myocardial enlargement in *D. melanogaster*. These pathological changes in the heart are related to systemic dysregulations, for example, in the context of energy supply and utilization via insulin-TOR signaling and via hexosamine flux and autonomic changes in tissues (e.g., adipose tissue, heart tissue). A reduction in lifespan is also evident under these conditions [22,135].

Compared to obesity in *D. melanogaster*, obesity in mammals is associated with myocardial fat deposition and fibrosis, diastolic and systolic dysfunction, arrhythmias, hypertension, heart failure and ischemic and hemorrhagic stroke, having a negative impact on life expectancy. However, an obesity paradox is described, in which obesity provides an advantage in a pre-existing pathology such as coronary artery disease and improves survival [235].

The obesity paradox, which suggests a beneficial effect of accumulated fat, also seems to be observable in the model organism, *D. melanogaster*. For example, fat droplets in the glial niche decrease ROS during oxidative stress and prevent the oxidation of PUFAs, protecting glial cells and neuroblasts [236].

However, in a clear difference from mammals, *D. melanogaster* exhibits an open circulatory system with hemolymph, as opposed to the mammalian, closed blood circulation. Thus, pathological abnormalities such as the deposition of atherosclerotic plaques cannot be studied in *D. melanogaster*.

Metabolic consequences of obesity can also involve the liver, which has led to increased incidences of human liver diseases worldwide, such as nonalcoholic fatty liver disease and hepatocellular carcinoma [237].

Invertebrates such as *D. melanogaster* demonstrate an evolutionarily conserved liver function, with the fat body and oenocytes exhibiting glycogen storage and lipid processing equivalent to the livers of vertebrates. In *D. melanogaster*, 22 genes of lipid metabolism orthologous to human genes are expressed in oenocytes. The lipid content in oenocytes is regulated by the lipase BRUMMER and LIPID STORAGE DROPLET-2 of the fat body, which are homologous to ATGL and perilipin (PLIN) in mammals [238].

The fruit fly reveals many concordant genetic and metabolic traits of liver diseases, which enables the investigation of modifying factors and pathogenesis. Metabolic disruption of lipid and glucose metabolism, for example, via the alteration of the expression of *dTOR*, *FASN*, sterol regulatory element-binding protein (*SREBP*) and *Dilps* in response to an HSD and HFD, negatively affects tissues and organs [239]. The structure of the fat body and oenocytes can be visualized by staining to detect irregular arrangement, altered sizes or altered quantities of stored lipid droplets. HSD and HFD feeding increases the number and size of lipid droplets in the fat body, similar to the situation in mammalian steatosis [23,129]. These initial conditions can chronically lead to the development of liver inflammation, cirrhosis or carcinoma [239].

Furthermore, obesity and comorbidities (e.g., diseases, hypertension) are major risk factors for the development of kidney disease.

A function homologous to that of the mammalian kidneys is found in nephrocytes and Malpighian tubules in the model organism, *D. melanogaster*. Hemolymph is filtered in nephrocytes, and urine is released into the hindgut via the Malpighian tubules following the absorption of required substances such as water, iron and organic metabolites. Additionally, the molecular pathways are conserved in the fruit fly, which enables the investigation of genetic changes such as renal development, cell differentiation or renal pathologies [233,240].

HSD consumption results in metabolic dysfunctions in *D. melanogaster* that predispose the flies to insulin resistance and nephrocyte failure. In response to HSD feeding, the expression of *sticks and stones* (*Sns*), regulated by the hexosamine biosynthesis pathway and the Polycomb gene complex, declines, affecting the function of the immunoglobulin superfamily protein Sns, a homolog of mammalian nephrin. Sns and Kirre, homologous to Neph1, constitute part of the nephrocytic diaphragm and are essential for the ultrafiltration of hemolymph in garland nephrocytes. Structural changes in nephrocytes decrease ultrafiltration, causing, for example, the development of insulin resistance and increased proteinuria, indicating diabetic nephropathy parallel to that in mammals [241,242].

Additionally, HSD consumption leads to morphological alterations in Malpighian tubules involving reduced anterior and posterior diameters and a decreased density of microvilli in the brush border, resulting in a reduction in fluid secretion rate. Oxidative stress is also elevated, and inflammatory processes are activated in Malpighian tubules, leading to renal tubular dysfunction [140]. In mammals, increased oxidative stress and inflammatory processes are similarly linked to the development of chronic kidney disease [243].

In addition, the pathology of nephrolithiasis can be studied in *D. melanogaster*. HSD consumption increases the uric acid concentration in the hemolymph and enhances acidification to increase the formation of uric acid stones via purine catabolism [137]. Similarly, an increased acid load in the kidneys (e.g., due to increased endogenous synthesis), the insufficient availability of H^+^ buffers and a low urine pH promote the formation of renal stones in humans [244]. The formation of the most common type of calcium oxalate renal stones in humans [245] can also be induced in the fruit fly by direct administration of lithogenic substances, e.g., calcium oxalate or ethylene glycol, in the diet [246].

Moreover, obesity can negatively affect the reproductive capacity of males and females. At both the genetic and morphological levels, many similarities between *D. melanogaster* and mammals are present.

In male fruit flies, the main cell types (e.g., spermatogonial stem cells, spermatocytes, meiotic cells) and the steps of spermatogenesis in the testes are analogous to those in mammals [247]. Diet affects male fertility, where a high-carbohydrate and low-protein diet leads to improved short-term offspring productivity. Carbohydrates seem to maintain the energy supply level and ensure the production of sex peptides and ovulin in the seminal fluid, which affects reproductive success [248]. HFD feeding in males causes a downregulation of glycosidase (e.g., α-mannosidase) activity at the transcriptional level, which could directly affect gametogenesis, and consequently, sperm quality and function [159]. Similarly, HFD feeding in male rats induces obesity and adversely affects carbohydrate metabolism and testicular morphology, disrupting spermatogenesis [249]. Additionally, in men, correlations among obesity, comorbidities such as diabetes and reduced fertility caused by, for example, erectile dysfunction, a reduced semen quality or increased germ cell apoptosis can be observed [250].

The ovaries in female *D. melanogaster* have a cellular organization similar to that in mammals. Each follicle comprises 15 nurse cells and one oocyte enclosed by follicle cells. The ovaries of both fruit flies and mammals contain follicles at different stages of development and maturation [251].

HSD-induced obesity reduces ovary size in *D. melanogaster*, with the increased storage of TAGs and cholesterol and reductions in mature egg numbers [142] and fecundity [75]. In addition, increased levels of oxidative stress and alterations of the mitochondrial gene expression profile are observed in the ovaries [142].

A similar observation was made in obese women exhibiting altered oocyte morphology with increased cytoplasmic granularity [252]. The oocyte size is also reduced, and the resultant blastocysts contain reduced numbers of cells and higher TAG content [253]. Additionally, increased incidences of menstrual abnormalities (e.g., irregularity), polycystic ovarian syndrome, and ovulatory dysfunction and reduced spontaneous pregnancy rates are observed [254]. These findings are coupled with increases in oxidative stress and inflammation in the ovaries [255].

### 5.3. Lessons Learned from D. Melanogaster

The previous paragraph shows that central aspects of the *D. melanogaster* lipid metabolism such as FA synthesis and lipolysis were found to be evolutionarily conserved when compared to mammalian organisms. This raises the question whether the powerful fruit fly model can be used to identify novel genes, uncover novel aspects of lipid metabolism and help to develop novel anti-adipose strategies and treatments. Being a genetic model in the first place, it is no wonder that, in recent years, several genes that are involved in the regulation of the lipid metabolism were identified by forward and reverse genetic approaches in the fruit fly model first, before they were validated in mammals. For example, a *D. melanogaster adp* (*adipose*) loss-of-function mutant exhibits increased fat storage [19]. Genetic analyses revealed that *adp* encodes a WD40/tetratricopeptide-repeat-domain protein (WDTC). The mammalian homolog WDTC1 is a component of a DDB1-CUL4-ROC1 (CRL4) E3 ligase and was later shown to suppress adipogenesis via the regulation of peroxisome, proliferator-activated receptor-γ activity [256,257]. Remarkably, *WDTC1* SNP variants were defined as human obesity markers [258].

Two large-scale RNAi screens revealed approximately 500 and 60 novel candidate obesity genes, respectively, of which a great proportion had a homolog in mammals [259,260]. Based on these results, subsequent mice studies unveiled the role of Hedgehog signaling in mammalian adipocyte differentiation [260] and the regulatory role of the universal, store-operated calcium entry pathway in lipid metabolism [261].

On the other hand, the *D. melanogaster* model was successfully used to verify the function of candidate obesity genes that were identified by human genome-wide association studies as loci associated with adiposity [262] and as rare variants by the exome sequencing of severely obese children [263]. Testing the physiological function of the homologous genes by RNAi in the fruit fly model led to the discovery of 26 and 4 genes, respectively, which had not previously been linked to human obesity [262,263]. 

Due to the abovementioned high degree of functional and/or evolutionary conservation of the nutritional physiology and the key pathways of energy metabolism, *D. melanogaster* is recognized as a screenable model for anti-obesity agents [264,265,266,267]. Although published in an unchronological order, the aqueous extract of the brown algae *Saccorhiza polyschides* was first identified for mitigating the delirious effects of HFD in *D. melanogaster* [267] before it was found to counteract diet-induced obesity in mice [268]. In a recent study, a hydroethanolic extract of *Lampaya medicinalis* was tested for anti-adipogenic effects in *D. melanogaster* and HepG2 hepatocytes, preventing HFD-induced TAG accumulation and an accompanied inflammatory response in both systems. Compared to in vitro screens, the use of the fruit fly has the advantage of covering pharmacological aspects, including bioavailability, the influence of the gut microbiota and biotransformation. Moreover, the employment of fruit flies allows a cheap and rapid in vivo evaluation without major ethical considerations. Hence, it has the potential to precede/replace rodent studies at early stages of compound screening/identification. Lastly, apart from diet-induced obesity models, drug screening is also feasible in a number of genetic obesity models.

## 6. Conclusions

**Figure 16:**
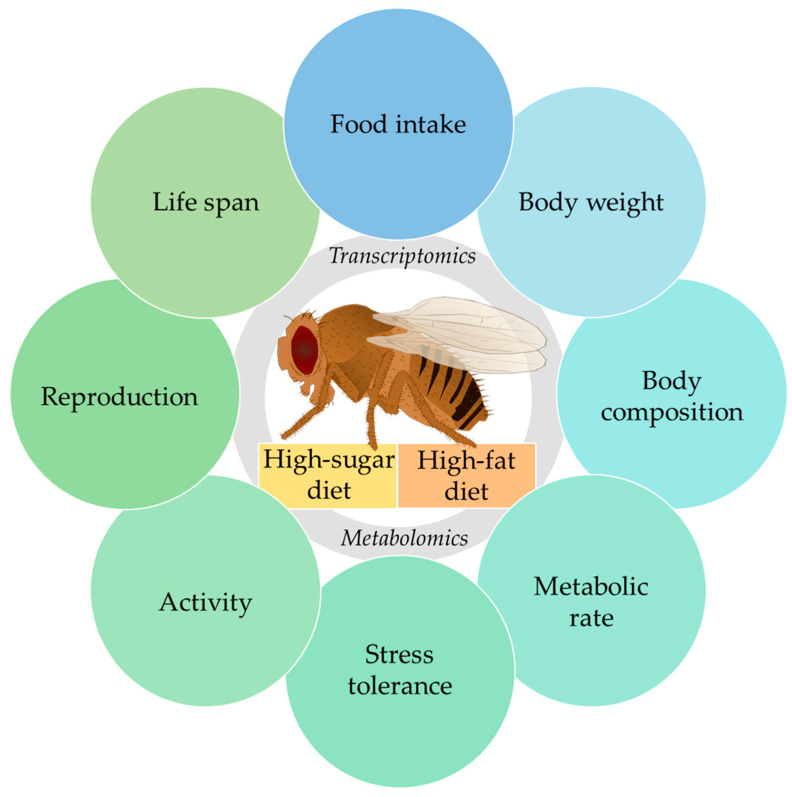
Overview of the methodological tool box that can be applied to study the responses of *Drosophila melanogaster* to high-sugar or high-fat diets from the molecular level to phenotypical traits.

Overall, a variety of different methods, such as the determination of body composition, food intake and metabolic rate, enable comprehensive phenotyping of the model organism, *D. melanogaster* (Figure 16). The diversity of methodologies allows the adaptation to different research questions, laboratory equipment, and time and financial frameworks. The structures of many organs, such as the digestive tract, and metabolic signaling pathways, such as the regulation of energy homeostasis via peptide hormones, are evolutionary and/or functionally conserved in *D. melanogaster*, making the fruit fly a well-suited model organism to study chronic diseases such as obesity and diabetes in a nutritional context from phenotypes to underlying cellular and molecular mechanisms. Furthermore, the influence of the parental diet on offspring, such as those in the F1 or F2 generation, can be studied.

Using *D. melanogaster* as a model organism has merits but also some limitations. First, not all organ and tissue structures of the fruit fly are functionally equivalent to those of humans and other mammalian species and some are even lacking. For instance, the fruit fly has only an innate immune system, which excludes studies of the response of the adaptive immune system to changing environmental factors such as microbial exposure.

In the future, there is a requirement for a standardization of dietary interventions for *D. melanogaster.* Terms such as “standard medium”, “cultivation medium”, “HSD” and “HFD” should be unified across laboratories in terms of the ingredients and their amounts. This will simplify the comparability of the study results of the different *Drosophila* research groups worldwide.

In summary, based on the diversity of methods for comprehensive phenotyping and its application in studies with dietary interventions, it can be concluded that *D. melanogaster* is a suitable and versatile model organism for the nutritional sciences.

## Figures and Tables

**Table 1:** Methodological overview of the determination of triacylglycerides (TAGs) with advantages and disadvantages.

Method	Advantages	Disadvantages
Dye staining (Nile red, BODIPY, Harris hematoxylin)	Cellular level: Determination of the number and size of lipid droplets	No quantification of total TAG content
Coupled colorimetric assays	Determination of the whole-body TAG content	No identification of the proportions of different glycerides
	Rapid determination	Removal of heads (due to colored eye pigment) recommended
	Large sample size	
Thin-layer chromatography	Proportions of TAG, diglycerides, fatty acids measurable	Extraction performed with organic solvents like chloroform or methanol
	Quantification of lipid classes available	Not compatible with the protocol shown in Figure 1
Liquid chromatography or gas chromatography/mass spectrometry	Specific and highly sensitive	High cost per measurement

**Table 2:** Advantages and disadvantages of methods used for the determination of food intake and food choice in *Drosophila melanogaster*.

Method	Advantages	Disadvantages
Dye labeling of the food	Solid medium Large fly groups	Indirect determination of food intake via marker Inaccurate results (fast dye accumulation) Flies must be killed for measurement
Consumption-excretion (Con-Ex); Excreta quantification (EX-Q)	Long-term measurement of food intake No killing of flies (EX-Q)	Overhead or horizontal position of food source (not suitable for all fly strains)
Radioactive labeling of the food	Sensitive Solid medium Large fly groups No homogenization of flies	Indirect determination of food intake via marker Underestimation (retention of radio-isotopes in digestive tract)
CApillary FEeder (CAFE) assay	Applicable for food intake and food choice Direct measurement of food intake No killing of flies Accurate and consistent	Overhead position of food source (not suitable for all fly strains) Only liquid medium
Proboscis extension	Simple setup Recording of individual flies High time resolution	Highly dependent on experimenter Limited time of application Not suitable for larger fly groups and longer time periods
MAnual FEeding (MAFE) assay	No interference of foraging and feeding start	Liquid medium in a capillary
Automated detection of food intake	Sensitive High resolution Applicable for food intake and food choice Recording of individual flies Simultaneous recording of feeding behavior of numerous flies Recording independent of experimenter	No standard conditions of maintenance Not suitable for larger fly groups Expensive in comparison to other methods
Fly Liquid-Food Interaction Counter (FLIC)	Short- and long-term observation Recording of movement pathways, food assessment, circadian feeding behavior Linkage with other hardware, e.g., shock-triggering component	Only liquid medium
fly Proboscis and Activity Detector(flyPAD)	Solid medium	No long-term observation

**Table 3:** Overview of dietary intervention studies regarding high-sugar diet (HSD) or high-fat diet (HFD) feeding in *Drosophila melanogaster*. The studies are ordered by release date.

Diet	Strain, Sex, Age	Time Period of Intervention	Assays (Number of Larvae or Flies per Treatment or n = Number of Treatments)	Outcomes	Publication
**HSD**					
Combination of different sugar and yeast ratios HSD 2.5–40% sugar 2.5–40% yeast 1.5% agar 1.8% fungicide	*yw; w^1118^; Canton-S* Males, females 1 d	Prefeeding 3 d 10% sugar/yeast 13 d, 26–40 d, 52–56 d	*Food intake* (80 females/50 males) *Body composition* TAG Proteins (80 females/50 males) *Lifespan* (>250 females) *Fecundity* (80)	*Food intake* ↑ *Body composition* TAGs ↑ Protein storage ↓ *Lifespan* ↓ *Fecundity* ↓ Amplification of effect with age	Skorupa et al. [75]
Control diet 5% sugar 10% yeast 2% peptone 1% agar HSD 34% sugar	*Canton-S* L3 larvae	Shortterm: 12 h or Longterm: Egg to L3 Wandering L3 stage	*Food intake* *Body size*, *body weight* (>15) *Body composition* Hemolymph: Glucose (>160) Trehalose (>15) Whole body: TAG Carbohydrates (>25 larvae, >30 flies) *Development* (*n* = 3) *Molecular analysis* qRT–PCR (*n* = 7) Western blot (*n* > 4) Lipid droplet measurement (>15) Microarrays	*Calorie intake* ↑ *Body size* ↓ *body weight* ↓ *Body composition* Glucose ↑ Trehalose ↑ *Development time* ↑ *Molecular response* Insulin resistance ↑ Lipid droplet size ↑	Palanker Musselman et al. [129]
Control diet 10,400 mM sucrose, fructose, glucose or trehalose 8% yeast 1.5% agar HSD 1000 mM sucrose, fructose, glucose or trehalose	Mix of two wild populations Females 5 d	Egg to 5 d adult females	*Body weight* *Development* *Stress assay* Cold tolerance assays: nonlethal chronic cold: 0 °C, 16 h (50) acute cold stress: −3.5 °C, 2 h (100) *Body composition* TAG (50) *Molecular analysis* Metabolic fingerprinting (90)	Results for sucrose: *Development time* ↑ *Stress tolerance* ↓ *Body composition* TAGs ↑ Sorbitol ↑ Sucrose ↑ Fructose ↑ Inositol ↑ Malate ↑ Xylitol ↑ Results for fructose: *Body weight* ↑ *Development time* ↑ *Stress tolerance* ↓ *Body composition* TAGs ↑ Malate ↑ Fructose ↑ Sorbitol ↑ Trehalose ↑ Inositol ↑ Results for glucose: *Body weight* ↑ *Stress tolerance* ↓ *Body composition* TAGs ↑ Valin ↑ Free phosphate ↑ GABA ↑ Sorbitol ↑ Inositol ↑ Maltose ↓ Results for trehalose: *Body weight* ↑ *Development time* ↑ *Stress tolerance* ↓ *Body composition* TAGs ↑ Sorbitol ↑ Trehalose ↑ GABA ↓ Galactose ↓	Colinet et al. [130]
Control diet 5% sucrose 10% yeast 2% peptone 1% agar HSD 34% sucrose	*w^1118^* L3 larvae Females	Virgin females 7 d	*Body weight* *Body composition* Whole body: Glucose Trehalose Glycogen (>150 larvae/>160 flies) Hemolymph: Glucose Trehalose (>160) *Molecular analysis* Real-time quantitative PCR (qRT–PCR, >10 pooled samples)	Females: *Body weight↓* *Body composition* Whole body: Trehalose ↑ Glycogen ↑ TAGs ↑	Buescher et al. [128]
Control diet 5% sucrose 10% yeast 2% peptone 1% agar magnesium sulfate calcium chloride HSD 34% sucrose	*w^1118^* Males After eclosion	3 weeks	*Body composition* TAG (12) Hemolymph: Glucose Trehalose (35–45) *Lifespan (50)* *Development* *Molecular analysis* Western blot (*n* = 10) Optical heartbeat analysis Fluorescence staining, imaging (*n* = 3)	*Body composition* TAGs ↑ Glucose ↑ Trehalose ↑ *Lifespan* ↓ *Development time* ↑ *Molecular response* Insulin resistance ↑ Hexosamine flux ↑ Heart: Irregular beating patterns ↑ Fibrillations ↑ Asystolic periods ↑ Heart contractility ↓ Heart TAGs ↑ Heart collagen ↑ Mortality ↑	Na et al. [135]
Paternal diet: Control diet 3% sucrose 1% yeast 3% soy flour 1.2% agar HSD 10 or 30% sucrose F1 flies: 1.8% yeast 1% soy flour 8% yellow corn meal 2.2% molasses 8% malt extract 1.2% agar	*w^1118^* Males 4–5 d	Parental flies 2 d on HSD Investigation of F1 flies 7 d post eclosion	*Body composition* TAG Glucose Trehalose (>15) *Wing size* (*n* > 3) *Food intake* CAFE (*n* > 3) *Metabolic rate* Respirometer (>15) *Development* (25) *Molecular analysis* Eye pigment (>15) Sperm dissection (25) and RNA isolation Fat bodies: Immunofluorescence (*n* = 7) RNA sequencing Bioinformatic analysis	*Body composition* TAGs ↑	Öst et al. [143]
Control diet 5% sucrose 10% yeast 2% peptone 1% agar HSD 34% sucrose	*w^1118^* Females 1 d	Virgin females 7 d on HSD F1 adult males 14 d on HSD	*Body length* (5) *Ovarian area*, *ovarian length,* *ovarian width* (83–93) *Fecundity* Stage 14 eggs (40) *Development* (>9/development stage) *Ovarian composition* TAG Glucose Cholesterol (>250) *Molecular analysis* Insulin sensitivity (>30) qRT–PCR: Heads (40) Ovaries (>48) L3 male larvae (40) Protein carbonylation (>30)	*Ovarian area* ↓ *ovarian width* ↓ *Fecundity* Egg number ↓ *Ovarian composition* TAGs ↑ Cholesterol ↑ *Molecular response* Insulin resistance ↑ Insulin production ↓ Oxidative stress ↑ Alteration of mitochondrial gene profile	Brookheart et al. [142]
Control diet 5% sucrose 10% yeast 1.5% agar HSD 40% sucrose	*w^1118^* Females 2 d	1–3 weeks, followed by 1 week on control diet	*Lifespan* (403) *Molecular analysis* RNA sequencing (RNA-seq) qRT–PCR Western blot S-adenosylmethionine ELISA 4′,6-Diamidino-2-phenylindole staining (50–90 nuclei)	*Lifespan* ↓ Detrimental effects in mid-life and late life *Molecular response* FOXO phosphorylation ↑ Insulin-like signaling ↑	Dobson et al. [136]
Control diet (cornmeal/yeast fly medium) HSD +1 M sucrose	*w^1118^* Females 5–7 d	5–7 d	*Intestinal microbiome* Bacterial load (10) *Molecular analysis* Immunostaining assays *7*-Aminoactinomycin D (7-AAD) Dihydroethidium (DHE) staining (gut; *n* = 3) RNA sequencing	*Intestinal microbiome* Alteration in bacteria composition Growth rate ↑ Bacterial number and density ↓ *Molecular response* p-JNK levels ↑ lacZþ cells ↑ Downregulation of JAK/STAT Reactive oxygen species levels ↑ Cell death ↑ Intestine: Disruption of gut homeostasis Fat droplets ↑ Differentiation of intestinal stem cell (ISCs) ↑ Midgut diameter ↓ Disorganization of gut cells Disruption of gut cell membrane function	Zhang et al. [138]
Control diet 5% sucrose 8% yeast 2% yeast extract 2% peptone 1% agar HSD 34% sucrose (1 M)	*w^1118^* Females 3 d	3 weeks	*Lifespan* (945) *Food intake* *Body weight* (5) *Molecular analysis* Smurf (945) Alkaline phosphatase (5) Imaging of midgut (3) Immunohistochemistry of fly gut Intestinal and bacterial cell cultures	*Food intake* ↑ *Body weight* ↓ *Molecular response, pathology* Intestine: Gut permeability ↑ Intestinal alkaline phosphatase activity ↓ Gut length and diameter ↓ Broken and disorganized actin filaments in posterior midgut	Pereira et al. [139]
Control diet sucrose (0.15 M) cornmeal/yeast medium HSD sucrose (1 M)	*w^1118^* Post-eclosion L3 larvae	3–5 d	*Molecular analysis* Larval and adult phagocytosis (15–20) F-actin (5–10 larvae) Hemocyte (10 larvae) Immunostaining (lymph glands, fat bodies, 5–10 larvae) Terminal deoxynucleotidyl transferase dUTP nick end labeling (TUNEL) 7-AAD staining (10 larvae) qRT–PCR (10 larvae)	*Molecular response*, *pathology* Cell structure: Larval and adult phagocytosis ↓ Larvae: Disrupted filaments of plasmatocytes and F-actin cytoskeleton crystal cells ↓ Lamellocytes ↑ in lymph glands Immune system: activation of Toll and JNK signaling pathways TUNEL- and 7-AAD-positive cells in fat body ↑	Yu et al. [133]
Control diet 5% sucrose 7.5% white corn syrup 2% yeast 1% soy flour 7% cornmeal 1% agar HSD: 20 or 30% sucrose	*w^1118^* Males 2–5 d; 6–8 d (RNAi lines)	2 and 7 d	*Food intake* PER (22–61) FLIC (16–72) Optogenetic stimulation for FLIC Taste sensitivity *Body composition* TAG (24) Protein (3/replicate) *Molecular and neuronal analysis* Nile red staining (fat bodies of 3 flies) Sensillar electrophysiology Metabolomics (100 heads) Calcium imaging (26 brains) RNA sequencing Proboscis immunofluorescence qRT–PCR (40–50 heads)	*Food intake* PE ↓ over time Behavioral responses to food ↓ Sweetness sensitivity ↓ Meal duration and size over time ↑ Modification of satiety but not hunger *Body composition* Fat ↑ *Molecular and neuronal response* Taste neurons: Presynaptic responses over time ↓ Stimulation of hexosamine biosynthetic pathway (HBP) O-GlcNAc ↑ Activity O-GlcNAc transferase (OGT) ↑ Genes encoding proteins with neural function- and metabolism-related activity, e.g., glutaminase and carbonate dehydratase	May et al. [131]
Control diet 8% sucrose 0.5% yeast 2% agar 0.16% calcium chloride dihydrate 0.16% ferrous sulfate heptahydrate 0.8% sodium potassium tartrate tetrahydrate 0.05% sodium chloride 0.05% manganese chloride tetrahydrate 0.53% nipagin HSD +300 mM sucrose	*Canton-S*; *w^1118^* Males, females 1, 3, 5 weeks	4 d	*Body weight* *Body composition* TAG (>30) Hemolymph: Glucose Trehalose (>100) *Locomotion activity* Climbing (>100) Activity (50) Flight (>100) *Food intake* CAFE (>50) *Lifespan* (100) *Starvation* (50) *Molecular analysis* Bromoenol lactone treatment (100) Cytological analysis of muscle and adipose tissues Ultrastructural analysis of muscle (*n* > 3) qRT–PCR (*n* > 3) Western blot (10)	*Body weight* ↑ *Body composition* TAGs ↑ *Locomotion activity* Climbing ability ↓ Flight performance ↓ *Molecular response* Insulin resistance ↑	Villanueva et al. [134]
Control diet 7.5% white corn syrup 1.7% yeast 1% soy flour 7% cornmeal 0.7% agar 0.9% propionic acid 0.4% tegosept HSD +30% sugar	*w^1118^* Males 2–4 d	18–24 h fasting or HSD feeding (refeeding)	*Body composition* TAG (24–28) *Food intake* FLIC (30) Foraging (24–28) *Molecular analysis* Metabolomics (300 heads; 150 bodies) RNA sequencing (10 brains)	*Food intake* FLIC feeding interaction ↓ Motivation of feeding ↓ *Metabolic response* Citric acid cycle activity ↑ 1-Carbon metabolism ↓ *N*-acetyl-aspartate ↓ Kynurenine ↓ Glucose usage ↑ *Neuronal response* GABA ↑ Glutamate ↑ Choline ↑ *N*-acetylserotonin ↑ Alteration of neurochemicals *Metabolomics* Changes in 54/377 compounds (e.g., fumarate ↑, malate ↑)	Wilinski et al. [132]
Control diet 15 g sugar (0.15 M) 6 g yeast 17 g maize 1.5 g agar 1 mL propionic acid 1 g methyl-paraben HSD 1 M sucrose	*Oregon-R* Male 1 d	5, 10, 15, 20 and 25 d	*Lifespan* (200) *Body composition* Hemolymph: Glucose Trehalose (30) Whole body: TAG Protein (30) *Molecular analysis* Uric acid (30) Images Immunohistochemistry F-actin staining Mitochondrial staining Western blot (24–30) Advanced glycation end products (40) RNA sequencing of Malpighian tubules (18) Fluid secretion measurement in Malpighian tubules (30) Oxidative stress parameters 30) qRT–PCR (*n* = 3)	*Lifespan* ↓ *Body composition* Glucose ↑ Trehalose ↑ TAGs ↑ *Molecular response* ROS ↑ Superoxide dismutase ↑ Catalase ↑ Cleaved-caspase 3 Advanced glycation end products ↑ V-ATPase and (Na^+^/K^+^)-ATPase ↓ Drosophila aquaporin *Pathology* Malpighian tubules: Diameters of anterior and posterior tubules ↓ Density of brush microvilli ↓ Mitochondrial size ↓ Large junction protein discs ↓ Uric acid ↑ Fluid secretion rate ↓ *Gene expression* Upregulation of the expression of 3940 and downregulation of 1852 (DAVID GO and KEGG pathway terms, ortholog GO classes and KEGG pathways)	Rani et al. [140]
Control diet 5% sucrose 10% Brewer’s yeast 1.5% agar 0.3% nipagin 0.3% propionic acid HSD 20% sucrose (partly 30%, 40%)	*w^Dah^*; *w^1118^* Males, females 2 d	7 and 28 d Adjustment of setup: water source	*Lifespan* (120–165) *Food intake* FlyPAD CAFE PER (50) Con-Ex (50) *Body composition* TAG (32) Hemolymph Volume Glucose Trehalose pH (72–96) *Fecundity* (150) *Stress assay* High salt Oxidative stress Starvation (100–200) *Molecular analysis* Lipid staining (fat body) Fat body Insulin sensitivity Glucose uptake (*n* = 5) Protein glycation, carbonylation assay (*n* = 4–10) Intestinal function Smurf assay (20) Excreta analysis (*n* = 5) Tubule and rectal ampulla imaging Tubule secretion assay(50) Metabolomics of rectal ampulla stones (25 stones) Uric acid assay (30)	*Lifespan* Dose dependence ↓, rescued by water supplementation *Food intake* FlyPAD ↑ CAFE water intake ↑ Con-Ex ↑ *Body composition* TAGs ↑ Hemolymph trehalose ↑ *Fecundity* ↓ *Stress tolerance* Salt ↓ Starvation ↓ *Molecular response*, *pathology* Water imbalance ↑ Hemolymph volume ↓ Uric acid ↑ Glycation adducts ↑ Insulin resistance Intestine: Total number and size of deposits ↓ Oblong excreta ↑ Malpighian tubules: Dark deposits ↑ Tubule secretion rates ↓ Acidification ↑ Rectal ampulla stones (presence and severity) ↑ Uric acid in stones ↑ Dysregulation of purine biosynthesis	van Dam et al. [137]
Control diet 5% sucrose 6.7% yeast 6.7% cornmeal 0.5% agar HSD 20 or 35% sucrose	*Oregon-R* Males, females 5–6 d	10 d	*Body weight* (80) *Locomotion activity* Climbing assay (150) Phototaxis assay (150) *Body composition* Glucose (60) *Molecular analysis* Fluorescence microscopy (20–30 heads, 20 bodies) Scanning electron microscopy (30 heads) Transmission electron microscopy (30 heads)	*Body composition* Glucose ↑ *Molecular response* Akt phosphorylation ↑ Insulin pathway ↑ Retina and lamina: Cleaved caspase 3 ↑ *Pathology* Eyes: Deficit in light responsiveness ↑ Vision defects ↑ Ommatidia disorganization ↑ Enlarged photoreceptor cell bodies and rhabdomeres (volume ↑) Apoptosis ↑ Eye section: Autophagy ↑	Catalani et al. [141]
**HFD**					
Control diet Yeast Corn starch Molasses HFD +3, 7, 15 or 30% (*w*/*v*) coconut oil	*w^1118^* Females 10–15 d	Control diet prefeeding 5 d 2, 5 and 10 d (mainly 5 d)	*Body composition* TAG Glucose (>35) *Locomotion activity* Climbing (>150) *Molecular analysis* High-resolution video-microscopy of hearts (>22) Cardiac TAG (>90) Gene expression	*Body composition* TAGs ↑ (dose-dependent) *Locomotion activity* Climbing ↓ *Molecular response,* *pathology* Heart: Pathological alterations Fat storage ↑ Contraction strength ↓ Conduction blocks ↑ Dysregulation of insulin-TOR pathway Changes insulin/glucose homeostasis	Birse et al. [22]
Control diet 5% sugar 10% yeast 2% peptone 1% agar HFD: 14.1% “Crisco fat” (soybean and palm oil)	*Canton-S* L3 larvae	Shortterm: 12 h or Longterm: Egg to L3 Wandering L3 stage	*Food intake* *Body size*, *Body weight* (>15) *Body composition* Hemolymph: Glucose (>160) Trehalose (>15) Whole body: TAG Carbohydrates (>25 larvae, >30 flies) *Development* (*n* = 3) *Molecular analysis* qRT–PCR Western blot Lipid droplet measurement Microarrays	*Calorie intake* ↑ *Body size* ↓ *body weight* ↓ *Body composition* Glucose ↑	Palanker Musselman et al. [129]
Control diet yeast corn starch molasses HFD +5, 10 or 20% (*w*/*v*) coconut oil (mainly 20%)	*w^1118^*; *Canton*; *Oregon-R* Females 3 d	7 d	*Body composition* TAG (>85) Glucose (>60) *Food intake* CAFE (70) *Lifespan* (50) *Stress assay* Anoxia (>195) Cold (>210) Starvation (110)	*Body composition* TAGs ↑ Glucose ↑ *Lifespan* ↓ *Stress tolerance* Cold ↓ Anoxic ↓	Heinrichsen et al. [110]
Control dietyeast corn starch molasses HFD +30% (*w*/*v*) coconut oil	*w^1118^* Females 5–10 d	Prefeeding 5 d 2, 5 and 10 d (mainly 5 d)	*Body composition* TAG (36) *Heart analysis* TAG hearts (>200) Heart periods (>16) *Molecular analysis* qRT–PCR (20) Western blot (20) Immunocytochemistry (hearts)	*Body composition* TAGs ↑ *Heart pathology* Heart: Lipid droplets ↑ Cardiac function ↓ *Molecular response* Dysregulation PGC-1/spargel pathway Transcription factor *SREBP* *Gene expression* srl ↓	Diop et al. [23]
Control diet 10% glucose 7% corn meal 4% yeast 0.6% agar HFD +5% or 15% (*w*/*v*) lard	*w^1118^* Males, females L3 larvae 1 d	L3 larvae 60 h post-hatching 10 d	*Lifespan* (>325) *Food intake* CAFE (>20) *Body composition* Larvae: Oil red O staining *Molecular analysis* Malondialdehyde measurement (>40) RT–qPCR (lipid metabolism-related genes, >10)	*Lifespan* Median lifespan ↓ *Body composition* Fat content of larval fat body ↑ *Molecular response* Malondialdehyde ↑ *Gene expression Acox57D-d* ↓ *Acsl* ↓ *fabp* ↓ *CCT1* ↓	Kayashima et al. [8]
Control diet 8% fructose 10% yeast 2% polenta 0.8% agar HFD +6.3 or 15% (*w*/*v*) lard	*w^1118^* Males	10, 20, 30, 40 d	*Lifespan* (>170) *Food intake* Feeding assay (>15) *Body composition* Glucose Trehalose (12) *Molecular analysis* Insulin sensitivity (9) Thin-layer chromatography (40) qRT–PCR Confocal microscopy (>10) Bacterial infection (4–5) Fluorescence-activated cell sorting (90) Oil Red O staining of FACS-sorted plasmatocytes (>35 cells) Plasmatocyte gene expression arrays (>5000 plasmatocytes) Plasmatocyte cell death	*Lifespan* ↓ *Body composition* TAGs ↑ Glucose ↑ Trehalose ↑ *Molecular response, pathology* Insulin sensitivity ↑ Muscle, midgut: 10xSTAT92E-GFP ↑ *Gene expression* Whole genome: *ap-1* ↑ *Fox* ↑ *ATF-CREB binding* *sites* ↑ *upd3* ↑	Woodcock et al. [124]
Control diet 10% sugar 10% yeast 1.5% agar 1% Tween 80 (*w*/*v*) HFD +2% (*w*/*v*) palmitic acid	*Canton-S* Males 3 d	Prefeeding 3 d 7 and 14 d	*Lifespan* (100) *Locomotion activity* Climbing (200) *Sensory perception* Odor stimulation (15) T-maze (200) Electrophysiological recordings (15) *Molecular analysis* q-RT–PCR Gene expression (*n* = 3)	*Lifespan* ↓ *Locomotion activity* Climbing ↓ *Sensory perception* Olfactory sensitivity: 8 out of 10 aromatic compounds ↓ Changed reaction to aromatics *Molecular response* Changes in olfactory and nutrient-related signaling pathways *Gene expression* Antennas: *DmOrco* ↓	Jung et al. [146]
Control diet 0.75% sucrose 1.3% yeast 6.5% cornmeal 0.7% agar HFD +3, 7, 15 or 30% (*w*/*v*) coconut oil (mainly 15%)	*Canton-S* Females Post eclosion	During development or only in adults	*Body length* (3% HFD; 100) *Locomotion activity* Activity level *Courtship* Mating success Courtship latency Courtship index Mating latency Competition (25) Courtship song recording (10) *Development* (3% HFD) *Molecular analysis* Cuticular hydrocarbon (40) GC/MS analysis (40) Metabolic rescue (30)	*Body length* ↓ (females) *Locomotion* *activity* ↓ *Courtship* Courtship time of females ↓ Male courtship latency ↓ *Molecular response* Dienes ↓ Monoenes ↓	Schultzhaus et al. [149]
Control diet 10% sugar 10% yeast 2% agar HFD +30% (*w*/*v*) coconut oil	*w^1118^* Females 2 d	5 weeks	*Lifespan* (>200) *Locomotion activity* Climbing (>100) *Body composition* TAG (40) *Heart analysis* Heart contraction (30) *Molecular analysis* qRT–PCR (10)	*Lifespan* ↓ *Locomotion activity* Climbing ↓ *Body composition* Whole-body TAGs ↑ *Heart analysis* Cardiac TAGs ↑ Heart rate ↑ Fibrillation ↑ Fractional shortening ↓ *Gene expression* *dSir2* ↓	Wen et al. [148]
Control diet 8% sucrose 0.5% yeast 2% agar 0.16% calcium chloride dihydrate 0.16% ferrous sulfate heptahydrate 0.8% sodium potassium tartrate tetrahydrate 0.05% sodium chloride 0.05% manganese chloride tetrahydrate 0.53% nipagin HFD +5% (*w*/*v*) coconut oil	*Canton-S*; *w^1118^* Males, females 1, 3, 5 weeks	4 d	*Body weight* *Body composition* TAG (>30) Hemolymph: Glucose Trehalose (>100) *Food intake* CAFE (>50) *Starvation* (50) *Lifespan* (100) *Locomotion activity* (50) Flight (>100) Climbing (>100) *Molecular analysis* Bromoenol lactone treatment (100) Cytological analysis of muscle and adipose tissues Ultrastructural analysis of muscle (>3) qRT–PCR (>3) Western blot (10)	*Body composition* TAGs ↑ Hemolymph Glucose ↑ Trehalose ↑ *Lifespan* ↓ *Locomotion activity* Climbing ability ↓ Flight performance ↓ *Molecular response* Phospho-Akt ↑ *Pathology* Muscles: Actin-containing myofibrillar and intramuscular ectopic fat ↑ Changed intramuscular lipid infiltration Mitochondrial deformation	Villanueva et al. [134]
Control diet yeast corn agar HFD +20% (*w*/*v*) coconut oil Partly: +5, 10 or 20% (*w*/*v*) coconut oil Partly: +coconut oil, soybean oil or lard	*Oregon-R; Orgon-S;* 2 control stains Virgin female flies	5–6 d Starvation 36 h	*Body composition* Whole body: TAG Cholesterol (18) Hemolymph: TAG Cholesterol (40) *Locomotion activity* (>46), *food intake* MAFE (29) FLIC (>38) BARCODE feeding (>48) Video-recording-based food seeking *Metabolic rate* Respirometer (>60) *Neuronal analysis* Calcium imaging (*n* = 7–10) RNA-seq Single-cell RNA-seq (75) LC–MS/MS analysis Western blot (protein from fly heads) Dot blot (hemolymph) qRT–PCR Immunofluorescence staining (Drosophila S2 cells)	*Body composition* TAGs ↑ *Locomotion activity* Hyperactivity upon starvation ↑ Hyperactivity trough saturated fat ↑ Walking speed before food contact ↑ Latency before food contact ↓ *Neuronal response* Sensitivity of AKHR^+^ neurons ↑ AKHR ↑ Autophagy in AKHR^+^ neurons ↓ AMPK-TOR signaling for regulation of AKHR	Huang et al. [147]
Control diet 10% sugar 5% yeast 1.2% agar HFD +10 or 30% (*w*/*v*) coconut oil	*w^1118^* Females 1 d post-eclosion (virgins) 4–5 d (mated)	1–3 weeks	*Food intake* CAFE (>24) *Lifespan* (>124) *Locomotion activity* Climbing (>8) Activity (>46) *Body weight* *Body composition* TAG Protein Glucose Glycogen (>30) *Fecundity* (>19) *Cold stress assay* (>80) *Molecular analysis* Immunostaining (brain, crops, heart, muscle, >8) qRT–PCR	Difference between virgin and mated females Results for 30% HFD: *Food intake* CAFE ↑ *Lifespan* ↓ (concentration-dependent) *Locomotion activity* Climbing ↓ Locomotion ↓ (virgins) *Body weight* ↓ *Body composition* Glucose ↑ (5 d) and ↓ (after 3 weeks) TAGs ↑ (5 d) and ↓ (after 3 weeks) (mated flies) *Fecundity* ↓ *Stress tolerance* Faster recovery (cold) *Molecular response* Akh ↑ (virgins) *Pathology* Crop size ↑	Liao et al. [144]

Acronyms: ↑ = Increased or upregulated; ↓ = Decreased or downregulated.

**Table 4:** Overview of dietary intervention studies regarding changes in transcriptome following high-sugar diet (HSD) or high-fat diet (HFD) feeding in *Drosophila melanogaster*. The studies are ordered by release date.

Diet	Strain, Sex, Age	Time Period of Intervention	Method(Tissue)	Outcomes(Process, e.g., Genes)	Publication
**HSD**					
Control diet 5% sugar 10% yeast 2% peptone 1% agar HSD 34% sugar	*Canton-S* Males L3 larvae	Shortterm: 12 h or Longterm: Egg to L3 Wandering L3 stage	Microarray (whole body)	Results short-term intervention: *Genes altered* 823 genes *Genes upregulated* Glucose transport (*Uggt*) Fatty acid (FA) synthesis (*FASN*) Trehalose synthesis (*Treh*) Triacylglyceride (TAG) storage (TAG lipases) Glycogenolysis (*GlyP*) Pentose phosphate shunt (*G6PD*) *Genes downregulated* Glycolysis (*Hex*) Results long-term intervention: *Genes altered* 309 genes *Genes upregulated* Glucose transport (*Uggt*) FA synthesis (*FASN*) Trehalose synthesis (*Treh*) TAG storage (TAG lipases) Gluconeogenesis (*Pepck*) β-oxidation (Acetyl-coenzyme A (acyl-CoA) dehydrogenases) Insulin signaling pathway (*dFOXO*)	Palanker Musselman et al. [129]
Control diet 5% sucrose 10% yeast 1.5% agar HSD 40% sucrose	*w^1118^* Females 2 d	1–3 weeks, followed by 1 week on control diet	RNA sequencing (whole body)	*Genes altered* 6435 genes *Genes upregulated* Insulin signaling pathway (*Dilp6*) *Genes downregulated* Insulin signaling pathway (*dFOXO*) Histone-modifying enzymes (*Acf*, *D12*, *egg*)	Dobson et al. [136]
Control diet 3% sucrose 4% inactive yeast 2.6% cornmea l0.8% agar 1.5% tegosept 0.3% propionic acid HSD 20% sucrose	*Oregon R-C* Females 2 d	7 d	RNA sequencing (heads)	*Genes upregulated* 164 genes Peptide metabolism Carbohydrate metabolism Hexokinase-like proteins Cytochrome P450 Mitochondrial activity Stress response (*hsp*) Infection defense, antimicrobial peptide production (*Dpt*) Apoptotic processes (*Decay*) *Genes downregulated* 69 genes DNA binding Transcription Cytochrome P450 function Kinase activity *heimdall* (*CG4500*)	Hemphill et al. [152]
Control diet 2% sucrose 22% cornmea l3% wheat germ 0.1% powdered milk 0.1% salt 0.1% soybean flour 0.1% rye flour Nipagin Lyophilized yeast HSD 30% sucrose	*Oregon-R* Males, females Eggs on control diet/HSD	7 d	RNA sequencing (whole body)	*Genes upregulated* 2695 genes Nuclear regulation processes Ribosome biogenesis target of rapamycin Mitogen-activated protein kinase Extracellular signal-regulated kinases (*ERKs*) Insulin signaling (*Dilps7*, *8*) *Genes activated from zero*: 3% Muscle structure Developmental growth Tissue development *Genes downregulated* 1136 genes Development muscle cells (*Actin*) Generation of precursor metabolites, enzymes and components of key energy pathways (*Pyk*, *kdn*, *ATPsynC*) Insulin signaling pathway (*Dilp6*, *p53* (*isoform A*)) *Genes knocked down*: 4% Cell structure Development	Loreto et al. [151]
**HFD**					
Control diet yeast corn starch molasses HFD 20% (*w*/*v*) coconut oil	*w^1118^* Females 3–5 d	7 d	Microarray (whole body)	*Genes upregulated* 1030 genes Immune response Carbohydrate binding Iron transport Extracellular secretion Amino acid metabolism Glycerophospholipids metabolism Sugar degradation Waste metabolites degradation *Genes downregulated* 297 genes *CG9510*	Heinrichsen et al. [156]
Control diet 3% sucrose 4% inactive yeast 2.6% cornmea l0.8% agar 1.5% tegosept 0.3% propionic acid HFD 20% (*w*/*v*) coconut oil	*Oregon R-C* Females 2 d	7 d	RNA sequencing (heads)	*Genes upregulated* 197 genes Peptide metabolism Carbohydrate metabolism Chitin metabolic processing Metabolic functions Stress response (*hsp*) Infection defense, immune function (*CecA1*, *AttA*) Antimicrobial peptide production (*Dpt*) Apoptotic processes (*Decay*) *Genes downregulated* 36 genes Nutrient storage Signal transmission Pyridoxal phosphate function *heimdall* (*CG4500*)	Hemphill et al. [152]
Control diet 10% sucrose 10% yeast 5% cornmea l1% agar 3% tegosept 0.3% propionic acid HFD 20% (*w*/*v*) coconut oil	*w^1118^* Females 2–7 d	7 d	Microarray (heads)	*Genes upregulated* 59 genes Odorant binding proteins (*Obp*, *OS9*) Odorant receptor coreceptor (*Orco*) Nucleotide metabolism (*Gfat1*, *Pepck2*, (*gogo*, *caps*) Stress responses (*CG2837*, *to*, *Cyp4e3*) *Genes downregulated* 132 genes DNA replicationMitosis Cell cycle regulation RNA binding Neurogenesis Oxidative stress (*TotC*, *hsp26/27*) Neuronal level (*CycA*, *brat*, *osk*, *pum*, *orb*, *tun*, *RnrS*, *CG3699*, *CG10962*) Neurophysiological mechanisms (*Fsn*, *lola*, *tyf*) Fat storage (*dob*)	Rivera et al. [157]
Control diet Food mix (New Horizon Foods) HFD 20% (*w*/*v*) coconut oil	*w^1118^* Males, females 3 d	7 d	RNA sequencing (heads, whole body)	Results for males: *Genes altered* Whole body: 464 genes Head: 143 genes *Genes upregulated* Whole body: Antioxidant capacity (*Cyp4e3*) Hyperphagic behavior (*to*), Lipid homeostasis (*Cyp4e3*, *CG5953*) *Genes downregulated* Whole body: Glycoside hydrolases (*LManIII–VI*) Heads: Stress, immune response (*TotA*, *TotC*, *TotM*) Fat body: Lipid homeostasis (*Lsp2*, *Gnmt*) Results for females: *Genes altered* Whole body: 330 genes Head: 93 genes *Genes upregulated* Whole body: Antioxidant capacity (*Cyp4e3*) Hyperphagic behavior (*to*), Lipid homeostasis (*Cyp4e3*, *CG5953*) Head: Lipid metabolism (*Mtpα*, *yip2*, *CG12262*) *Genes downregulated* Fat body: Lipid homeostasis (*Lsp2*, *Gnmt*)	Stobdan et al. [159]
Control diet 4% sucrose 2% yeast 4% cornmea l1% agarose 1% parahydroxybenzonate 1% propionic acid HFD 20% (*w*/*v*) coconut oil	*Canton-S* Males, females 5–10 d	7 d	RNA sequencing (whole body)	*Genes upregulated* 112 genes Long-chain fatty acid synthesis (*FASN2*) *Genes downregulated* 91 genes Results for males: *Genes upregulated* Molecular chaperone (*CCT1*) Oxidative stress response (*Sod1*, *Sod2*) *Genes downregulated* FA synthase (*FASN1*) Unsaturated FA synthesis (*Desat1*) *Drosophila* insulin-like peptide signaling pathway (*tobi*) Immune response (*Dro*, *DptA*) Results for females: *Genes upregulated* Unsaturated FA synthesis (*Desat1*) Glucose metabolism, gluconeogenesis (*sug*) Oxidative stress response (*Sod1*, *Sod2*) *Genes downregulated* *Drosophila* insulin-like peptide signaling pathway (*tobi*) Glucose metabolism, gluconeogenesis (*Amy-p*, *Amy-d*) Immune response (*Dro*, *DptA*) Egg production (*Vm26Aa*, *psd*)	Azuma et al. [162]

**Table 5:** Overview of dietary intervention studies regarding changes in metabolome following high-sugar diet (HSD) or high-fat diet (HFD) feeding in *Drosophila melanogaster.* The studies are ordered by release date.

Diet	Strain, Sex, Age	Time Period of Intervention	Method	Outcomes	Publication
**HSD**					
Control diet 10,400 mM sucrose, fructose, glucose or trehalose 8% yeast 1.5% agar HSD 1000 mM sucrose, fructose, glucose or trehalose	Mix of two wild populations Females 5 d	Egg to 5 d adult females	Gas chromatography/mass spectrometry	Results for sucrose: *Metabolite level* *increased* Sucrose Fructose Sorbitol Malate Results for fructose: *Metabolite level* *increased* Fructose Trehalose Malate Results for glucose: *Metabolite level* *increased* Whole-body metabolite concentrations Sorbitol Inositol Amino acids (e.g., valine, leucine, isoleucine) *Metabolite level* *decreased* Maltose Gamma-amino-butyric acid (GABA) Results for trehalose: *Metabolite level* *increased* Sorbitol Trehalose Amino acids (e.g., valine, leucine, isoleucine) *Metabolite level* *decreased* Galactose GABA	Colinet et al. [130]
Control diet 1% corn syrup 4% yeast 4% malt extract 7% cornmeal 1% soy flour 0.5% agar 0.4% propionic acid 0.8% tegosept HSD 18% corn syrup	*w^1118^*, split ends (Spen)-depleted *D. melanogaster* Males, females Larvae 22–24 h	First to late third instar larvae	Liquid chromatography/mass spectrometry	Results for *w^1118^*: Metabolic change: 4% of the analyzed metabolites were changed Results for Spen-depleted flies: Metabolic change: 7% of the analyzed metabolites were changed *Metabolite level* *decreased* L-carnitine	Gillette et al. [168]
Control diet 0.5% sugar 3% yeast 0.6% agar 5% cornmeal nipagin propionic acid HSD 18% and 30% sucrose	*w^1118^*; *D. melanogaster* with mild mitochondrial pyruvate carrier (MPC) 1 deficiency Males 15 d	15–25 d	^1^H Nuclear magnetic resonance spectroscopy	Results for *w^1118^*: *Metabolite level* *increased* Fructose Glucose-6-phosphate Proline *Metabolite level* *decreased* Leucine Malate Citrate Results for MPC1 deficient flies: *Metabolite level* *increased* Glycogen Fructose Glucose Glucose-6-phosphate Glycine Proline β-alanine	Simard et al. [166]
Control diet 5% sugar 10% yeast 2% peptone 1% agar HSD 34% sucrose	*w^1118^* Males, females 1 d	3–5 weeks	Ultra-high performance liquid chromatography/mass spectrometry/mass spectrometry	Results for fat body: *Metabolite level increased* (10 lipids) Diglycerides (DAG) Triacylglycerides (TAG) Saturated fatty acid, monounsaturated fatty acid (FA) substituents (e.g., lauric acid, myristic acid, palmitic acid) Relative proportion of double bonds Even:odd chain ratios Plasmenyl TAGs (DAGEs) Other plasmenyl, plasmanyl species Palmitate *Metabolite level* *decreased* 110 lipids Odd-chain esterified FAs Results for hemolymph: *Metabolite level* *increased* (33 lipids) DAG TAG Glycerolipids Even:odd chain ratios Saturated FA substituents DAGEs Lyso-phosphatidylcholines *Metabolite level* *decreased* (131 lipids) Odd-chain esterified FAs Phospholipid Unsaturated substituents Ceramides Sphingomyelin Other plasmenyl, plasmanyl species Results for heart: Altered: 44 lipids *Metabolite level* *increased* DAG TAG Chained TAGs Chained DAGEs Glycerolipids Palmitate *Metabolite level* *decreased* Odd-chain esterified FAs Odd-chain substituents PUFA substituents Other plasmenyl, plasmanyl species	Tuthill et al. [169]
**HFD**					
Control diet yeast corn starch molasses HFD 20% (*w*/*v*) coconut oil	*w^1118^* Females 3–5 d	7 d	Gas chromatography/mass spectrometry	*Metabolite level* *increased* Total fatty acids (e.g., palmitate, oleate, stearate) Lactate Pyruvate Urea Uric acid Acetyl-coenzyme A (acetyl-CoA) *Metabolite level* *decreased* Fumarate Malate α-ketoglutarate Numerous amino acids	Heinrichsen et al. [156]
Control diet Cornmeal-molasses food HFD 3% (*w*/*v*) coconut oil	16 different cultured genotypes Larvae First instar	First to late third instar larvae	Liquid chromatography/mass spectrometry Gas chromatography/mass spectrometry	*Metabolite level* *increased* Medium-chain FAs (caproate, caprylate, caprate, laurate) Dicarboxylic FAs (decanedioate, dodecanedioate) Monohydroxy FAs (3-hydroxysebacate, 3-hydroxydecanoate) Glucose-6-phosphate Fructose-6-phosphate Citrate Dipeptides	Oza et al. [171]
Control diet 1% light corn syrup 4% yeast 4% light malt extract 7% yellow cornmeal 1% soy flour 0.5% agar 0.4% propionic acid 0.8% tegosept HFD 15% (*w*/*v*) coconut oil	*w^1118^*; split ends (Spen)-depleted *D. melanogaster* Males, females Larvae 22–24 h	First to late third instar larvae	Liquid chromatography/mass spectrometry	Results for *w^1118^* *Metabolic change*: 11% of the analyzed metabolites were changed Results for Spen-depleted flies: *Metabolic change*: 24% of the analyzed metabolites were changed *Metabolite level* *decreased* Acetylcholine L-carnitine	Gillette et al. [168]
Control diet 0.5% agar 0.6% sugar 3% yeast 5% cornmeal nipagin propionic acid HFD 20% (*w*/*v*) coconut oil	*w^1118^* Males 10 d	10 d	Nuclear magnetic resonance spectroscopy	*Metabolite level* *increased* 25 metabolites 8 amino acids (serine, valine, tyrosine) Glycogen Glucose Glucose-6-phosphate Pyruvate Citrate Fumarate Succinate Malate Cytosolic nicotinamide-adenine dinucleotide Acetyl-CoA	Cormier et al. [172]

## Data Availability

Not applicable.

## Abbreviations

7-ADD	*7*-Aminoactinomycin D
Acetyl-CoA	Acetyl-coenzyme A
4EBP	eIF4E-binding protein
AKHR	Adipokinetic hormone receptor
AKT	Protein kinase B
AMPK	Adenosine monophosphate-activated protein kinase
ATGL	Adipose triglyceride lipase
BCA	Bicinchoninic acid
CAFE	CApillary FEeder
CCA	Coupled colorimetric assay
Con-Ex	Consumption-excretion
DAG	Diacylglycerol
DAM	Drosophila Activity Monitoring
dFOXO	Forkhead Box O
DHE	Dihydroethidium
Dilp	Insulin-like peptide
EB, EC, EE	Enteroblast, enterocytes, enteroendocrine
ERK	Extracellular signal-regulated kinase
EX-Q	Excreta quantification
FA	Fatty acid
FAS	Fatty acid synthase
FLIC	Fly Liquid-Food Interaction Counter
flyPAD	Fly Proboscis and Activity Detector
GABA	Gamma-amino-butyric acid
GFP	Green fluorescent protein
GO	Glucose oxidase
HBP	Hexosamine biosynthetic pathway
HFD	High-fat diet
HK	Hexokinase
HSD	High-sugar diet
Hsl	Hormone-sensitive lipase
ISC	Intestinal stem cell
JAK	Janus kinase
JNK	c-Jun *N*-terminal kinase
LC/MS; GC/MS	Liquid chromatography or gas chromatography/mass spectrometry
LSD	Low-sugar diet
MAG	Monoacylglycerol
MAFE	Manual feeding
MARK	Mitogen-activated protein kinase
NADH	Nicotinamide-adenine dinucleotide
NADPH	Nicotinamide-adenine dinucleotide phosphate
O-GlcNAc	O-linked *N*-acetylglucosamine
OGT	O-GlcNAc transferase
PAT	Perilipin, ADRP, TIP47
PBT	Dulbecco’s phosphate-buffered saline with Triton X-100
PER	Proboscis extension response
PI	Preference index
PKA	Protein kinase A
qRT–PCR	Real-time quantitative PCR
RING	Rapid iterative negative geotaxis
SAM (ELISA)	S-Adenosylmethionine ELISA
SIK3	Serine/threonine-protein kinase 3
STAT	Signal transducer and activator of transcription
TAG	Triacylglyceride
TCA	Tricarboxylic acid
TLC	Thin-layer chromatography
TOR	Target of rapamycin
TUNEL	Terminal deoxynucleotidyl transferase dUTP nick end labeling
VLDL	Very-low-density lipoprotein
WDTC	WD40/tetratricopeptide-repeat-domain protein
